# Multiple aspartic proteases process PE and PPE proteins on the mycobacterial surface of pathogenic mycobacteria

**DOI:** 10.1016/j.jbc.2026.113450

**Published:** 2026-08-13

**Authors:** Aniek S. Meijers, James L. Gallant, Alexander Speer, Janneke Maaskant, Saara Hietamäki, Roy Ummels, Felix M. Paulußen, Tom N. Grossmann, Thang V. Pham, Sander R. Piersma, Connie R. Jiménez, Sergey Nejentsev, Wilbert Bitter, Coen P. Kuijl

**Affiliations:** 1 Medical Microbiology and Infection Control, Amsterdam UMC, Vrije Universiteit Amsterdam, Cancer Center Amsterdam, Amsterdam, The Netherlands; 2 Molecular Microbiology, Vrije Universiteit Amsterdam, Amsterdam, The Netherlands; 3 Medical Microbiology and Infection Control, Amsterdam UMC, Vrije Universiteit Amsterdam, Amsterdam Institute of Infection & Immunity, Amsterdam, The Netherlands; 4 Department of Chemistry and Pharmaceutical Sciences, Vrije Universiteit Amsterdam, Amsterdam, The Netherlands; 5 Proteomics Core Resource, Laboratory Medical Oncology, Amsterdam UMC, Vrije Universiteit Amsterdam, Amsterdam, The Netherlands; 6 Department of Medicine, University of Cambridge, Cambridge, United Kingdom; 7 Department of Molecular Cell Biology and Immunology, Amsterdam University Medical Centers, Amsterdam, The Netherlands

**Keywords:** *Mycobacterium*, aspartic proteases, cell surface, protein secretion, PE proteins, PPE proteins, cleavage motif

## Abstract

*Mycobacterium tuberculosis* is the world’s leading bacterial infectious agent. The absence of an effective vaccine and the rise of multidrug-resistant *M. tuberculosis* strains emphasize the need to identify novel targets for prevention and intervention strategies. Previously, the *Mycobacterium marinum* orthologue of the aspartic protease PecA (PE_PGRS35) was found on the bacterial cell surface, after secretion by the specialized Type VII secretion system. PecA was shown to be responsible for the processing of secreted PE_PGRS proteins, including itself. Both *M. marinum* and *M. tuberculosis* encode two additional predicted aspartic proteases with similar secretion domains (PE_PGRS16 and PE26). The roles of the *M. marinum* PecA, PecB, and PecC were studied using frameshift mutants generated by CRISPR-Cas9 technology. Numerous PecA substrates were identified using analysis of semi-tryptic peptides detected by proteomics of secreted, surface-associated protein fractions. The most abundant substrates are members of the PE and PPE protein families. In addition, analysis of semi-tryptic peptides revealed consensus cleavage sites. Interestingly, these cleavage sites were also situated within the Type VII secretion motif (YxxxD/E), thereby effectively removing the entire PE domain, further strengthening the notion that these domains function as secretion peptides. PecB and PecC were also predicted to process PE and PPE proteins, although to a lesser extent than PecA. Follow-up experiments revealed that PecC is involved in the processing of a small subset of the PPE protein family. Finally, our evidence indicates that PecA functions similarly in *M. tuberculosis*, as PecA is also responsible for cleavage of PE_PGRS proteins in this species.

*Mycobacterium tuberculosis*, the deadliest pathogen worldwide, still has a global impact on millions of people. An estimated 1.3 million deaths are caused by *M*. *tuberculosis*, and more than 10 million people suffer from active tuberculosis disease annually (1). One of the problems in understanding the virulence characteristics of this pathogen is that it is evolutionary only distantly related to most other bacterial pathogens, both Gram-positive and Gram-negative (2). This is reflected in the sparse number of shared virulence genes (3). On the other hand, mycobacteria have multiple genes that are unique to this bacterial family, and some of these have been implicated in virulence. For instance, approximately 10% of the *M. tuberculosis* genome codes for members of the PE and PPE protein families (2). PE and PPE proteins are named after the Proline-Glutamic acid (PE) or Proline-Proline-Glutamic acid (PPE) motif in their conserved N-terminal domains of ∼110 or ∼180 amino acids respectively. PE and PPE proteins can be further subdivided into a PE- or PPE-specific subgroup based on the presence of a specific C-terminal domain. The largest and most enigmatic group are the so-called PE_PGRS proteins, whose genes contain Polymorphic GC-rich Repetitive (DNA) Sequences (PGRS) that encode a domain with a variable number of GGAGGX repeats (4). PE/PPE proteins are mainly found in the culture supernatant or on the mycobacterial surface after translocation by one of the Type VII secretion systems (5, 6). Currently, the functions of the vast majority of PE/PPE proteins are unknown; however, some PE/PPE proteins are implicated to be involved in roles including virulence and nutrient uptake (7, 8, 9, 10, 11, 12, 13, 14, 15).

A subset of PE proteins contains domains with predicted functions. For instance, three putative aspartic proteases have been identified in *M. tuberculosis* that belong to the PE protein family (16). The *Mycobacterium marinum* orthologue of aspartic protease PecA, a PE_PGRS protein, has been shown to process PE_PGRS proteins, including itself and *M. tuberculosis* lipase LipY, on the surface of *M. marinum* (17). This processing event removes the N-terminal PE domain that is required for secretion. PecA activity is essential for full virulence, as the *M. marinum* strain lacking PecA (*ΔpecA*) showed moderate growth reduction in zebrafish larvae as compared to the wildtype strain (17). Both *M. tuberculosis* and *M. marinum* have two additional putative PE proteins with a C-terminal aspartic protease domain, PE_PGRS16, PE26, MMAR_2272 and MMAR_1538, respectively (16), but no proteolytic activity has been shown for these additional proteases.

In this study, we investigated the role of the PE aspartic proteases in pathogenic mycobacteria. Substrates were identified using label-free mass spectrometry of *M. marinum* surface fractions, and we demonstrated that these aspartic proteases process PE and PPE proteins in addition to the PE_PGRS subfamily. Finally, we confirmed PecA protease activity in *M. tuberculosis*, indicating a conserved function.

## Results

### Aspartic proteases of *M. marinum* and *M. tuberculosis*

Recently, we have described the activity of a surface protease in *M. marinum* belonging to the PE protein family and named it PecA (17). The *M. tuberculosis* PecA homologue and two paralogues, PE_PGRS16 and PE26, had previously been identified (16); however, their proteolytic activity are not yet characterized. Similar to PecA, both proteins consist of an N-terminal PE domain containing the Type VII secretion signal YxxxD/E (18), followed by a PGRS domain. Although PE26 is not annotated as a PE_PGRS protein, this protein does contain the GRPLI-like motif of PGRS proteins followed by a short GGAGGX repetitive amino acid motif that is similar to the PGRS domain of PecA. The AlphaFold2 model of both *M. tuberculosis* PE26 and its *M. marinum* orthologue MMAR_1538 predicts a poly-glycine II (PG_II_) sandwich domain that is similar to other PGRS domain predictions (19, 20).

**Figure 1:**
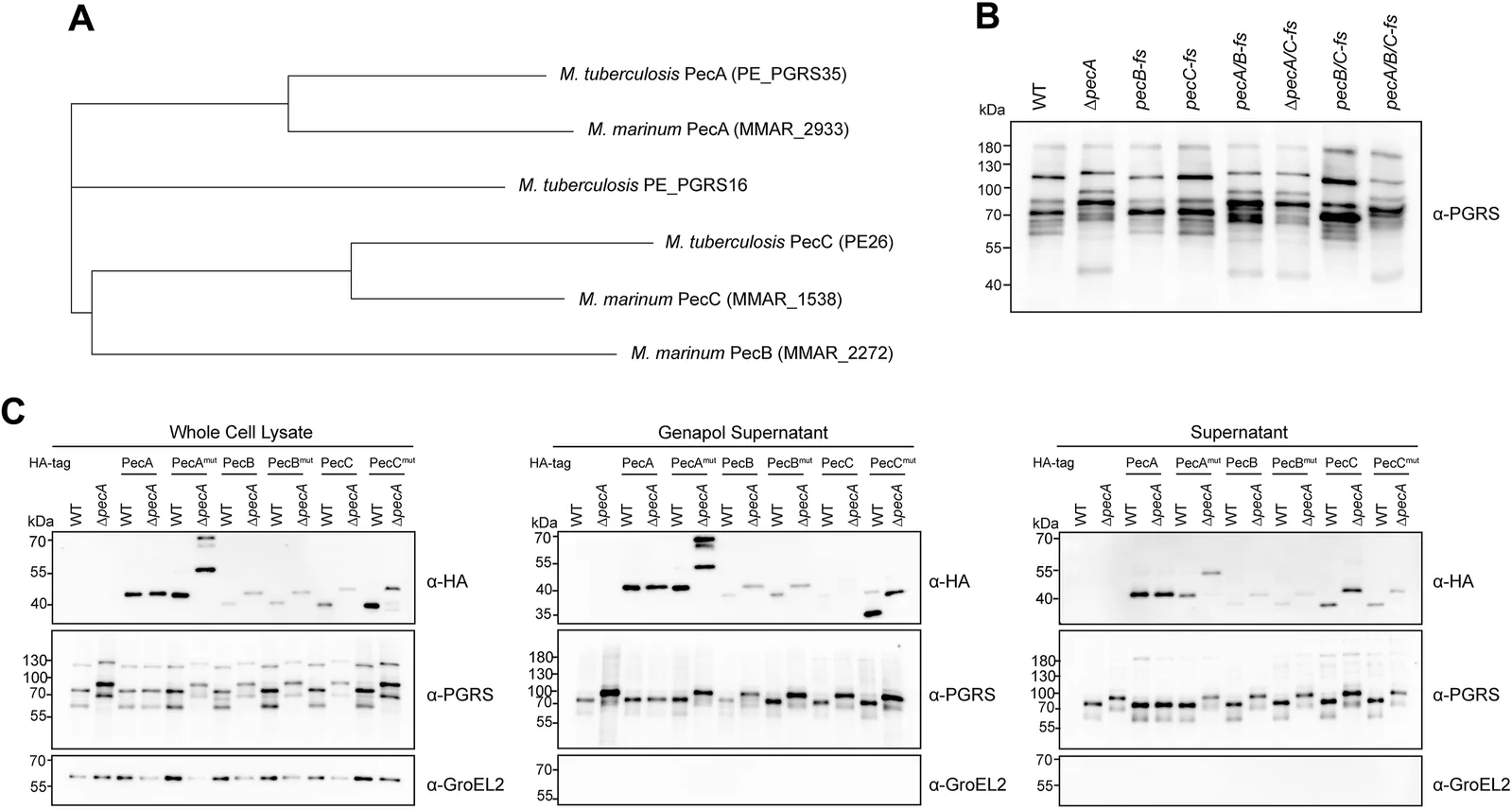
**Aspartic proteases of *M. marinum* and *M. tuberculosis.****A*, phylogeny of protease domains from *M. marinum* and *M. tuberculosis* aspartic proteases: PecA (MMAR_2933/PE_PGRS35), PecB (MMAR_2272), PecC (MMAR_1538/PE26), and PE_PGRS16 (ClustalW2). *B*, immunoblot of whole cell lysates from WT and single, double and triple *pecA/B/C* frameshift mutants grown in 7H9. PE_PGRS proteins were detected *via* anti-PGRS mAb 7C4.1F7 (0.2 OD/lane). *C*, expression of C-terminal HA-tagged proteases in *M. marinum* WT and *ΔpecA* strains. Fractions: whole cell lysate (WCL, 0.2 OD), supernatant (0.4 OD), and Genapol surface extract (0.4 OD). Anti-GroEL2 used as loading/lysis control. Representative of n = 3 (PecA/B) or n = 2 (PecC) independent experiments.

Both PE_PGRS16 and PE26 have a C-terminal domain that contains the DTG and DSG motifs characteristic of aspartic proteases. *M. marinum* strain M encodes not only PecA but also a close homologue of PE26 (MMAR_1538) and a third putative protease (MMAR_2272). However, this third putative protease shares only 38% to 45% amino acid identity with the other protease domains of the *M. tuberculosis* Pec proteases (Table S1). In addition, MMAR_2272 does not cluster with PE_PGRS16 when comparing the protease domains (Fig. 1*A*), therefore it does not appear to be an ortholog of PE_PGRS16. The crystal structure of the aspartic domain of PE_PGRS16 (16) and the AlphaFold2 predicted structures of the aspartic protease domains MMAR_2272, MMAR_1538, PE_PGRS16 and PE26 show that the aspartic motifs DTG and DSG within the protease are in close proximity to form a putative active site for protein cleavage (Fig. S1, *A* and *B*). Based on the similarity of the putative aspartic proteases to PecA, we assigned them to the PE cleavage protein family and designated them as PecB (MMAR_2272) and PecC (PE26/MMAR_1538), which will be used throughout this study. Protease domains of PecA and PecC share greater similarity to each other than to PecB, according to protein identity comparison (Table S1). PE_PGRS16 from *M. tuberculosis* is not further investigated in this study, but should be considered a putative fourth Pec protein. PE_PGRS proteins are restricted to species within the *M. tuberculosis* complex and a subset of other slow-growing mycobacteria, including the *M. marinum* and *Mycobacterium kansasii* branches. Because the putative Pec proteases belong to the PE_PGRS family themselves, they are not conserved in *Mycobacterium smegmatis* or in other fast-growing mycobacteria. However, some fast-growing mycobacteria do encode an aspartic protease that shares some similarity to the C-terminal domain of PecA, but these protease domains lack both the PE domain and the PGRS domain and therefore are not classified as PE_PGRS proteins.

### Generation of single, double, and triple *pecA/B/C* frameshift mutants by CRISPR-Cas9

To investigate the aspartic proteases of pathogenic mycobacteria, we generated single, double and triple mutants in *M. marinum* targeting *pecA* (MMAR_2933), *pecB* (MMAR_2272), and *pecC* (MMAR_1538). Building on our previous work (21), which demonstrated that single frameshift (fs) mutants can be efficiently generated using an anhydrotetracycline (ATc)-inducible *Streptococcus thermophilus* Cas9 (Sth1Cas9) system, we adapted this approach for sequential editing. We constructed the CRISPR plasmid pCRISPRx-Sth1Cas9-L5-HygR by replacing the kanamycin resistance cassette with a hygromycin resistance gene. By alternating these plasmids using gene-specific small guide RNAs (sgRNAs), we facilitated the iterative creation of frameshift mutants. This sequential strategy enabled the efficient generation of double and triple mutants, all of which were confirmed *via* PCR amplification and sequencing of the sgRNA target loci.

The sgRNA for targeting *pecA* was designed prior to the release of the CHOPCHOP tool (22), which predicts putative off-target activity toward the *pecB* gene for this sgRNA. Indeed, when we sequenced our *pecA* frameshift mutants for *pecB* mutations, all colonies tested harboured a 2 bp deletion at the predicted off-target site. We therefore utilized this *pecA* frameshift mutant strain as *pecA/B-fs* double mutant strain in this study. To investigate the role of PecA alone, we used the *ΔpecA* deletion mutant that was published previously (17). The *pecA/C and pecB/C* double and *pecA/B/C* triple mutants were established by generation of frameshift mutations in the *ΔpecA*, *pecB* and *pecA/B* mutants, respectively (Table S2). Finally, in all mutants, the CRISPR-Cas9 plasmid was replaced by the integrative plasmid pTdTomato-L5 (21). No differences in colony morphology were observed following disruption of *pecA*, *pecB* and/or *pecC* (data not shown). In addition, the growth rate of all mutants was similar to the wild-type (WT) strain as measured for 5 days during culture in 7H9 medium (Fig. S1*C*).

### PecA is responsible for processing of PE_PGRS proteins

To examine protease activity, we compared protein profiles of wild-type and *pecA*, *pecB*, and/or *pecC* mutants using Coomassie-stained SDS-PAGE gels. We observed no obvious differences, implying that the most abundant proteins that are visible on Coomassie-stained gels are not processed by PecA, PecB, or PecC (Fig. S1*D*). Previously, PecA was found to cleave many PE_PGRS proteins (17). To investigate whether PecB and/or PecC are also involved in the processing of PE_PGRS proteins, we used a monoclonal antibody that recognizes PGRS domains (5). As reported previously, in the Δ*pecA* mutant, the molecular weights of the detected PE_PGRS proteins appeared higher as compared to the WT strain (Fig. 1*B*). The increase in molecular weight of the PE_PGRS proteins detected was only visible in strains lacking PecA, whereas strains lacking PecB or PecC appeared identical to the wild-type PE_PGRS pattern. This indicates that PecA, but not PecB or PecC, is responsible for processing the observed PE_PGRS proteins.

### PecA processes aspartic proteases on the mycobacterial surface

Previously, it was established that PecA, expressed with a C-terminal myc-tag, is located on the surface of *M. marinum* and is capable of processing other PecA proteins *in trans* (17). To determine the localization of PecB and PecC, wild-type genes and catalytic inactive mutants (PecB^mut^ and PecC^mut^) were expressed with a C-terminal HA tag in WT and *ΔpecA* mutant strains. In addition, PecA and catalytic inactive PecA^mut^ were also cloned with a C-terminal HA-tag and introduced in WT and *ΔpecA* mutant strains. Secretion analysis confirmed that PecA cleaves itself, as full length PecA (∼54 kDa) was detected in the *ΔpecA* mutant strain complemented with PecA^mut^, whereas processed PecA (∼42 kDa) was found in strains expressing either endogenous PecA or overexpressed PecA (Fig. 1*C*). In line with previous results, PecA was detected on the mycobacterial surface following extraction with the mild detergent Genapol X-080 (Fig. 1*C*, Genapol Supernatant fraction). However, in this study, a small amount of PecA was also detected in the culture supernatant, suggesting that a minor fraction of PecA is released from the surface. In the *ΔpecA* mutant that expressed PecA^mut^, unprocessed 54 kDa full length PecA was detected, in addition to another prominent band with an apparent molecular weight of ∼70 kDa. The presence of this band suggests the occurrence of post-translational modifications or strong interactions with another protein, possibly a substrate.

Both PecB and catalytic inactive mutant PecB^mut^ were detected in the Genapol supernatant fraction, indicating that they are present on the mycobacterial surface, and in the culture supernatant (Fig. 1*C*). In WT strains, both wild-type and active site mutant PecB were detected with an apparent molecular weight of around 38 kDa, whereas PecB was detected around 43 kDa in *ΔpecA* mutant strains. Therefore, these data demonstrate that PecB is a substrate of PecA.

Finally, also both PecC and PecC^mut^ were detected on the mycobacterial surface and in the culture medium (Fig. 1*C*). Molecular weight of full-length PecC was predicted at ∼48 kDa, which can be detected in the *ΔpecA* mutant strains expressing PecC and PecC^mut^. In WT strains, PecC was present around 37 kDa, which shows that PecA is responsible for the processing of PecC.

### Identification of *M. marinum* aspartic protease substrates by label-free mass spectrometry

To identify additional PecA substrates, and to assess if PecB and PecC also function as active proteases, we performed label-free mass spectrometry. Surface proteins of *M. marinum* wildtype (WT), Δ*pecA* and *pecA/B/C* triple mutant cultures were extracted using the mild detergent Genapol X-080, digested with trypsin, and peptides were detected by LC-MS/MS (Table S3).

**Figure 2:**
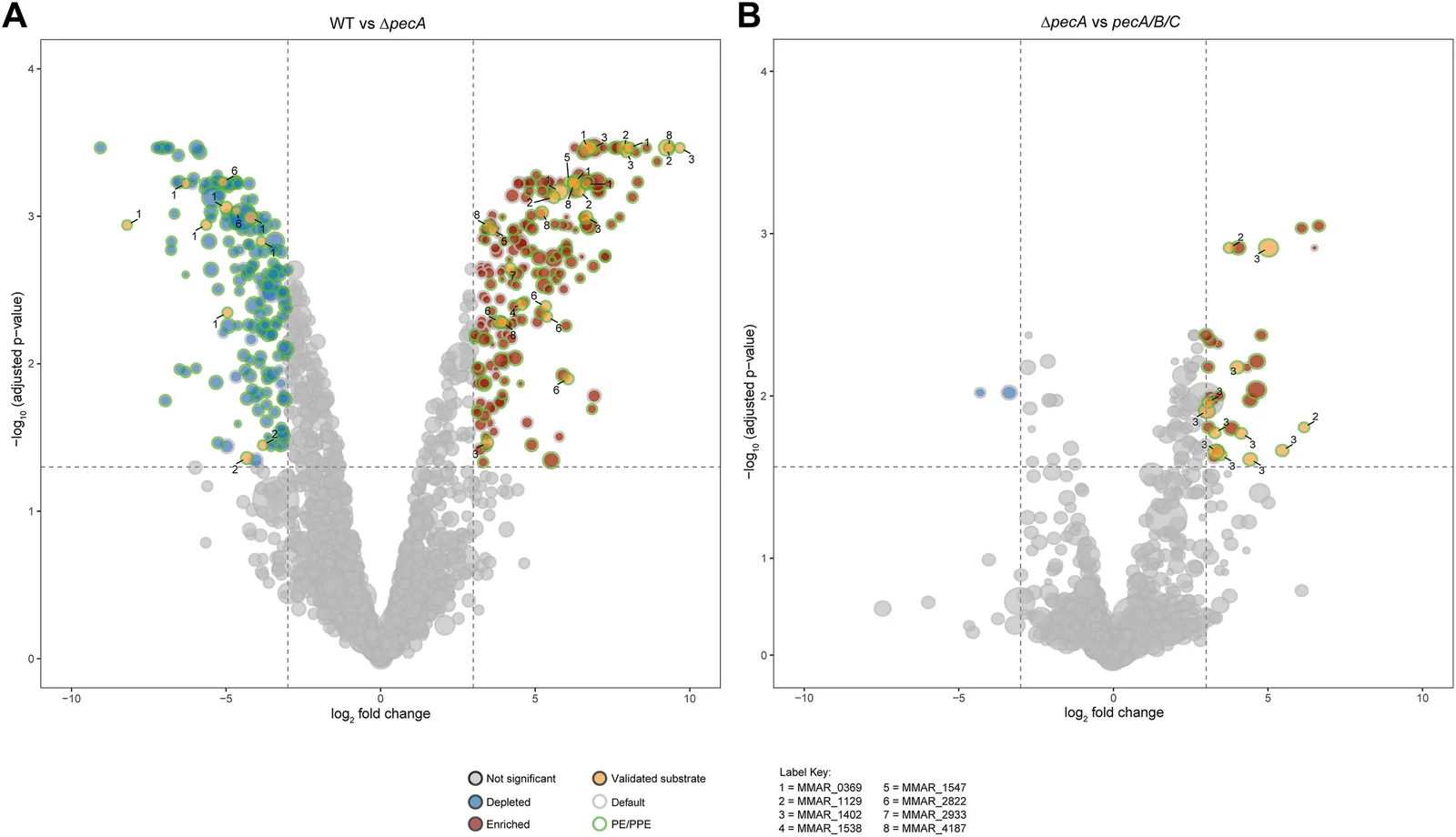
**Identification of protease-generated peptides by PecA and PecB/C.***A*, Volcano plots depicting all semi-tryptic peptides identified by proteomics of surface fractions of *M. marinum* wildtype compared to the Δ*pecA* mutant (*A*) and the Δ*pecA* mutant compared to the *pecA/B/C* triple mutant (*B*). Enriched semi-tryptic peptides (Table S3, log_2_ fold change of >3, adjusted −log_10_*p*-value (q-value) >1.3 (0.05)) of validated substrates are labeled with their corresponding gene name. The dot size is reflected by mean LFQ values. The data is representative of 3 independent biological replicates.

Putative PecA substrates were identified by enriched semi-tryptic peptides on the surface in the WT strain as compared to the Δ*pecA* mutant using a log_2_ fold change of 3 and q-value (adjusted *p* value) of 0.05 (Fig. 2*A*). This resulted in a list of 216 enriched semi-tryptic peptides (Table S3). As expected, several semi-tryptic peptides (63) belonging to 39 PE_PGRS proteins were enriched in the WT sample, as well as 14 peptides from 6 proteins of the PE family. Multiple (53) semi-tryptic peptides of 22 PPE proteins also appeared to be generated by PecA, as well as 55 other proteins that do not belong to the PE/PPE families (86 semi-tryptic peptides).

Similarly, putative PecB/C substrates were selected from semi-tryptic peptides enriched in the Δ*pecA* mutant over the *pecA/B/C* triple mutant (Fig. 2*B*), using the same criteria of a log_2_ fold change 3 and a q-value of 0.05. In total, 32 semi-tryptic peptides were enriched in the Δ*pecA* mutant compared to the *pecA/B/C* triple mutant. These enriched peptides belong predominantly to members of the PE/PPE protein families, with 18 semi-tryptic peptides belonging to 6 PPE proteins. In addition, 8 semi-tryptic peptides of 7 PE_PGRS proteins were enriched. This suggests that PecB and/or PecC are active as proteases and that they are, like PecA, also responsible for the processing of members of the PE_PGRS protein family. Moreover, 6 semi-tryptic peptides belonging to 5 proteins were enriched that did not belong to the PE/PPE protein families.

In this experiment, no tryptic or semi-tryptic peptides were detected for the PecB protein itself; therefore, it is unknown whether PecB is expressed or present on the mycobacterial surface under these growth conditions.

### Validation of PecA and PecB/C substrates

**Figure 3:**
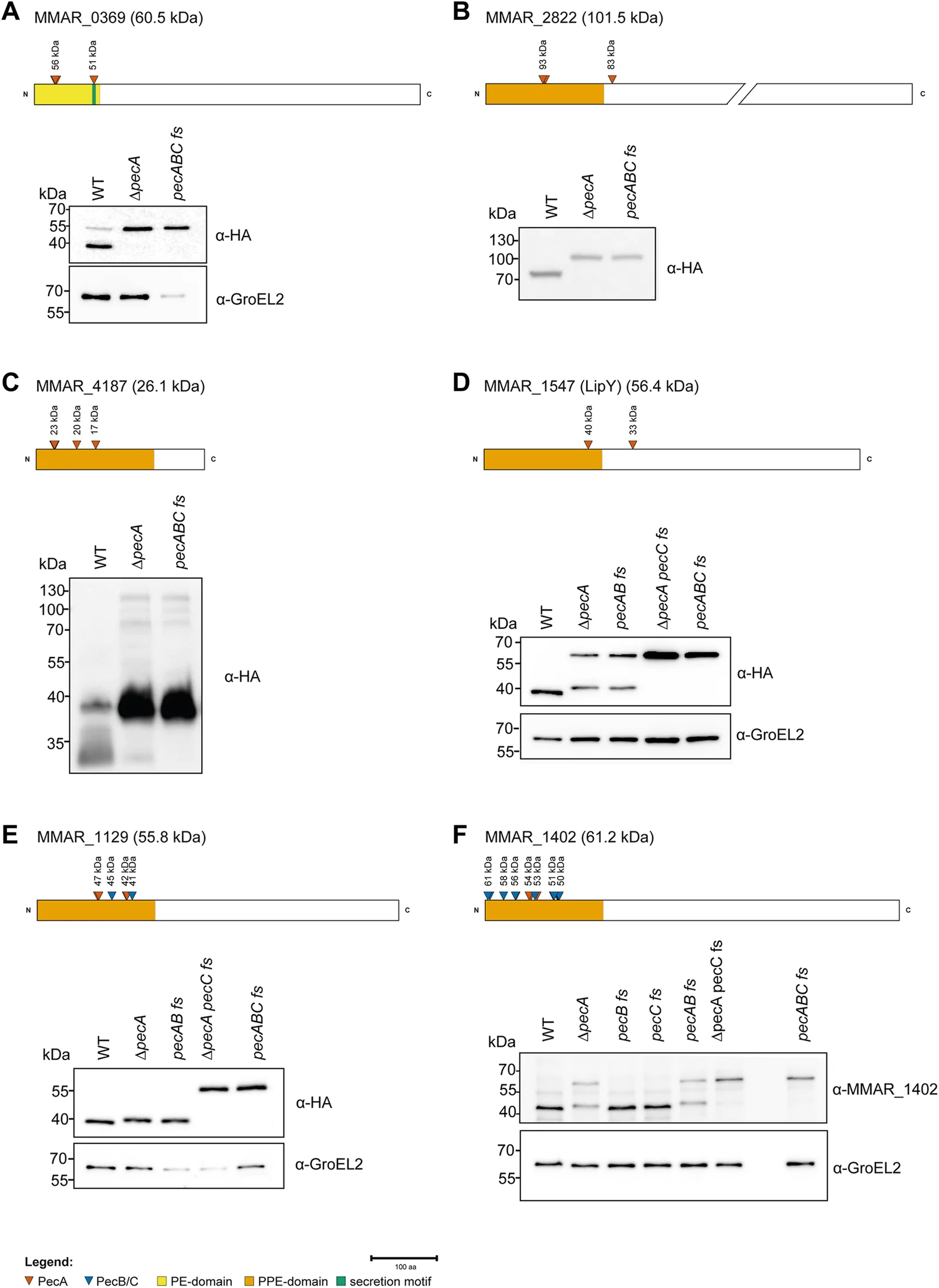
**Prediction and validation of substrate cleavage by PecA and PecB/PecC.***A–F*, schematic of observed cleavage sites and immunoblot validation of substrates. PE (*yellow*, ∼110 aa) and PPE (*orange*, ∼180 aa) domains are shown with secretion motifs (*green*). Cleavage sites identified by MS (Table S3) are indicated by *red* (PecA) and *blue* (PecB/C) triangles. Protein lengths and molecular weights (kDa) are shown to scale. For validation, (*A–E*) HA-tagged substrates were expressed in *M. marinum* WT and indicated *pec* mutants. Whole-cell lysates from 7H10-grown cells were analyzed by SDS-PAGE and anti-HA immunoblotting; GroEL2 or Ponceau S served as loading controls (Fig. S1*E*). *F*, MMAR_1402 was detected in 7H9-grown WT and *pec* mutants using a specific C-terminal polyclonal antibody.

Subsequently, we selected several putative PecA and PecB/C substrates to validate the mass spectrometry results. This selection includes members of the PE/PPE families listed in Table S3, and a schematic overview of their predicted cleavage is depicted in Figure 3. To validate whether PecA cleaves PE proteins without a PGRS domain, we studied MMAR_0369, a PE protein with a C-terminal α/β hydrolase domain (23). A gene coding for an HA-tagged version was introduced in WT, Δ*pecA* and the *pecA/B/C* triple mutant strains. Indeed, C-terminally HA-tagged MMAR_0369 was observed as a processed ∼39 kDa band on immunoblot in the WT strain, whereas a larger MMAR_0369 product (∼55 kDa) was detected in the Δ*pecA* and *pecA/B/C* triple mutant (Fig. 3*A*). These data confirm that PecA cleaves MMAR_0369 and releases the C-terminal hydrolase domain from the PE domain.

In addition to PE proteins, several PPE proteins were also suggested to be cleaved by PecA. We investigated this for MMAR_2822 and MMAR_4187 with a C-terminal HA-tag. MMAR_2822 was indeed processed by PecA as full-length (∼100 kDa) was detected in the Δ*pecA* and *pecA/B/C* triple mutant strains, whereas MMAR_2822 was detected ∼80 kDa in the WT strain, which corresponds with the most C-terminal semi-tryptic peptide identified (Fig. 3*B*). The PPE protein MMAR_4187 appeared partially cleaved in the WT strain (observed ∼30 kDa), whereas predominantly full-length protein (visible at ∼39 kDa, at a higher molecular weight than predicted) was detected in the Δ*pecA* mutant and the *pecA/B/C* triple mutant strains, which indicates that PecA cleaves MMAR_4187 (Fig. 3*C*). A minor fraction of cleaved MMAR_4187 was still visible in the *pecA* mutant but completely absent in the triple mutant. This suggests that PecB or PecC is responsible for this residual processing, although the cleavage was not identified by mass spectrometry.

A third PPE protein that we tested is *M. marinum* LipY, a triacylglycerol lipase. In *M*. *tuberculosis*, LipY is produced with an N-terminal PE domain, and is a validated *M. marinum* PecA substrate (17). However, although *M. marinum* LipY (MMAR_1547) is highly similar and has the same function (24), the N-terminal PE domain is exchanged for a PPE domain in this species. We evaluated the processing of *M. marinum* LipY using an HA-tagged version (Fig. 3*D*). Indeed, LipY was cleaved in the WT strain, while the apparent full-length LipY was detected in the Δ*pecA* mutant, as expected. However, a cleaved version of LipY remained visible in the Δ*pecA* mutant. We therefore also introduced the same construct in our other mutants, which showed *M. marinum* LipY is also cleaved by PecC, although there was no enriched semi-tryptic peptide found in the corresponding proteomics dataset.

PecB and/or PecC were predominantly predicted to cleave several members of the PPE family. The PPE protein MMAR_1129 was a predicted substrate of PecB or PecC as well as PecA. C-terminally HA-labelled MMAR_1129 was processed in the WT strain as it resolved with an apparent molecular weight of ∼39 kDa (Fig. 3*E*). A slightly higher band (∼40 kDa) was visible in both the Δ*pecA* and *pecA/B-fs* mutant strains, whereas full-length MMAR_1129 was only visible in the *pecA/C* and *pecA/B/C-fs* mutant strains. These results are in partial agreement with predicted cleavage patterns by semi-tryptic analysis and show that this protein is cleaved by PecA and PecC, although it should be noted that MMAR_1129 cleaved by PecC was predicted to be smaller than MMAR_1129 processed by PecA, which was not observed on immunoblot.

The PPE protein MMAR_1402 was also identified as a putative PecB or PecC substrate, as ten semi-tryptic peptides mapping to this protein were generated by PecB or PecC. Additionally, MMAR_1402 was predicted to be a PecA substrate. Endogenous MMAR_1402 was detected using a polyclonal antibody that recognizes its C-terminal domain, allowing the predicted cleavage patterns to be visualized by immunoblot (Fig. 3*F*). In the WT and the *pecB* and *pecC* single fs mutant strains, a band of ∼45 kDa was observed, representing the processed substrate. A slightly higher band, as well as a potential full-length (∼61 kDa) protein, was visible in the Δ*pecA* and *pecA/B* fs mutant strains. Finally, only unprocessed MMAR_1402 was observed in the *pecAC* and *pecA/B/C* mutant strains. These data confirm that both PecA and PecC cleave MMAR_1402. It should be noted that while the MMAR_1402 version cleaved by PecC was predicted to be smaller than the form processed by PecA, this difference was not observed on the immunoblot.

### Identification of PecA cleavage site

**Figure 4:**
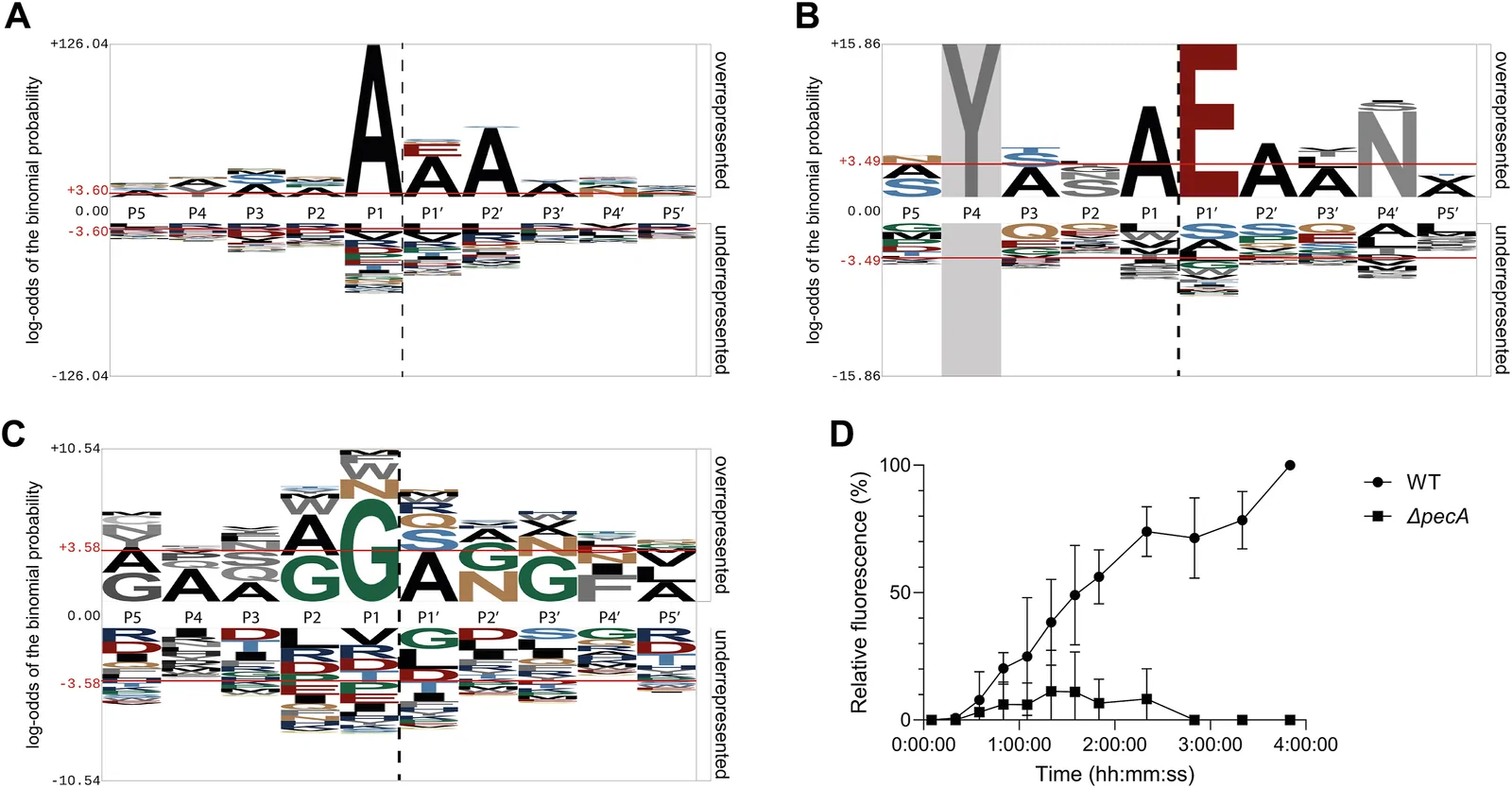
**PecA and PecB/C cleavage site predictions.** Analysis of enriched semi-tryptic peptides for prediction of PecA and PecB/C cleavage windows by pLogo (44). *A*, predicted cleavage sites of PecA by comparison of cleavage windows of *M. marinum* WT vs Δ*pecA* mutant strains using all *M. marinum* FASTA CDS as background. *B*, prediction of PecA cleavage site within enriched semi-tryptic peptides belonging to PE proteins, with fixed tyrosine at P4, with *M. marinum* PE and PPE FASTA CDS as background. *C*, predicted cleavage site of PecB/C following comparison of cleavage windows *M. marinum* Δ*pecA* and *pecA/B/C* mutant strains using all *M. marinum* FASTA CDS as background. Identified sites are shown following analysis of 199, 20 and 31 ten-residue (P5–P5′) cleavage sites for Figure *A*, *B* and *C*, respectively. The dotted lines indicate the predicted cleavage sites. *D*, fluorescent measurement of *M. marinum* WT and Δ*pecA* mutant strains following incubation with the FRET peptide FP53, which contains an N-terminal quencher DABCYL, a previously identified PecA cleavage site (17) and a C-terminal FITC. Relative fluorescence was calculated relative to WT at the final time point for each experiment. Means and standard deviations are presented based on two biological replicates.

To characterize the substrate specificity of PecA and PecB/C, we generated sequence logos based on ten-residue (P5–P5′) cleavage sites identified through semi-tryptic peptide enrichment. The PecA recognition motif was defined by comparing WT and Δ*pecA* strains, while the PecB/C motif was investigated by comparing the Δ*pecA* mutant against the *pecA/B/C* triple frameshift mutant. These motifs were statistically weighted against a background consisting of all *M. marinum* coding sequences or only PE and PPE family proteins. Analysis of PecA cleavage sites revealed a high prevalence of alanine at the P1 position (88%), indicating a dominant preference for peptide bond hydrolysis on the carboxyl side of alanine. This site was frequently flanked by alanine or glutamic acid residues at the P1′ position (Fig. 4*A*).

Sub-analysis focused specifically on the PE protein family confirmed the high frequency of alanine at the P1 position and identified a 29% occurrence of tyrosine at the P4 position (Fig. S1*F*). Upon fixing tyrosine at P4, the canonical Type VII secretion system signal motif (YxxxD/E; (18)) became apparent, specifically the YxxxE variant (Fig. 4*B*). A predominant alanine at the P1 position was also observed when analyzing cleavage sites within the PPE protein family (Fig. S1*G*). Collectively, these findings suggest that PecA predominantly cleaves alanine at the carboxyl side, including a specific subset of Type VII secretion signals, aligning with our previous study (17).

Next, all PecB/C cleavage windows derived from enriched semi-tryptic peptides were analyzed, obtained by comparing the *ΔpecA* mutant with the *pecA/B/C* triple frameshift mutant. Although a clear consensus motif could not be identified, these data suggests that PecB/C appears to preferentially cleave after a glycine (Fig. 4*C*).

Previously, 2 *M. marinum* PecA cleavage sites were identified within the heterologously expressed *M. tuberculosis* lipase LipY (17). To investigate whether the proteolytic activity of PecA is present at the surface, we used a FRET-based reporter assay. We incubated *M. marinum* WT and *ΔpecA* mutant strains with a peptide (FP53) containing the previously identified LipY cleavage site QVPPFA|AAQ (17) with an N-terminal quencher DABCYL and a C-terminal fluorophore FITC (Fig. 4*D*). This identified *M. tuberculosis* LipY cleavage site is similar to the cleavage site found in *M. marinum* in our proteomics dataset (ADPPFA|ASQ). Processing of the FRET peptide was monitored by the resulting increase in fluorescence. Incubation with WT cells resulted in a clear fluorescent signal, whereas no fluorescence was observed after incubation with the *ΔpecA* mutant strain, indicating that PecA is active at the surface (Fig. 4*D*).

### PecA degrades only PE_PGRS proteins from the same cell

Almost all PE_PGRS proteins detected by immunoblotting in strains expressing PecA on the cell surface appear to be processed (17). In addition, we previously observed that a minute amount of *M. tuberculosis* PecA is required to obtain full processing of PE_PGRS proteins in *M. marinum*, since expression of *M. tuberculosis* PecA from an inducible promoter did not require induction for full complementation (17). Taking the extracellular localization of PecA and proteolytic activity toward a peptide substrate into account, we examined whether PecA is able to cleave substrates of neighboring cells. This could have importance during mycobacterial culture or during infection of the host. To study PecA processing activity beyond its own surface, we performed a co-culture of WT and *ΔpecA* mutant strains at 1:1, 1:10, 1:100 and 1:1000 ratios for 20 to 24h before whole-cell lysates were prepared. We observed that all PE_PGRS proteins appeared cleaved in the wild-type strain, whereas half of the PE_PGRS proteins were processed after culturing wild-type and *ΔpecA* mutant strains at a 1:1 ratio, which was most clearly visible for the PE_PGRS protein detected ∼115 kDa (Fig. S1*H*). Moreover, a minor fraction of the PE_PGRS proteins (around 1/10th) appeared cleaved in the 1:10 ratio co-culture. No PE_PGRS proteins appeared processed in the 1:100, 1:1000 and *ΔpecA* mutant conditions, indicating that PecA on the surface of wild-type cells does not efficiently cleave PE_PGRS proteins beyond its own cell surface.

### PecA activity in *M. tuberculosis*

**Figure 5:**
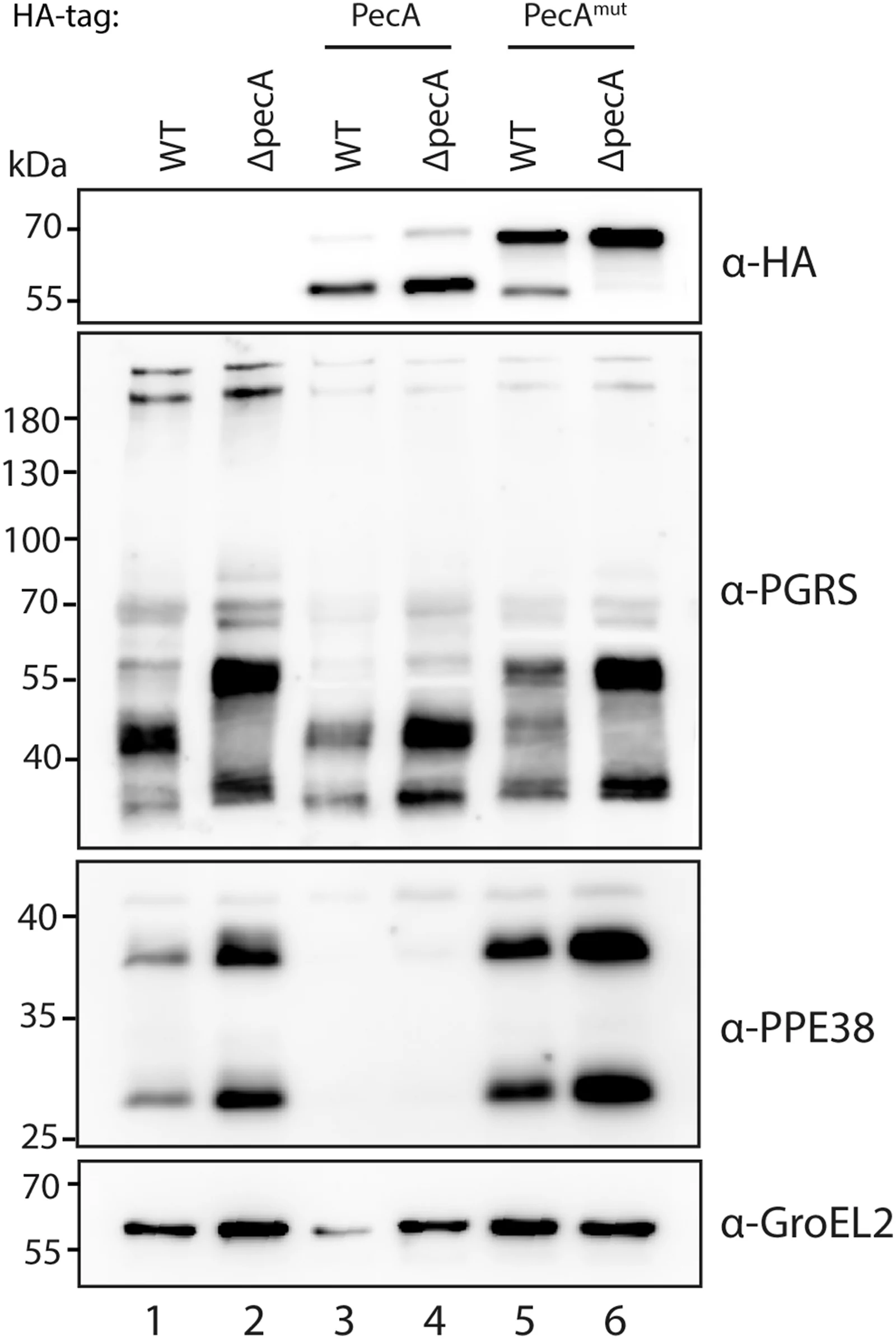
**PecA activity in *M. tuberculosis*.***M. tuberculosis* PecA and active site mutant PecA^mut^ with C-terminal HA tag were expressed in CDC1551 WT and *ΔpecA* mutant strains, induced on 7H10 plates containing 0.5 mM theophylline and cells were harvested from plate. Expression of HA-tagged proteins, PE_PGRS proteins, PPE38 and GroeL2 (loading control) was detected by immunoblotting. Representative data is shown of 3 colonies tested per strain.

Finally, to investigate if PecA is active in *M*. *tuberculosis*, *pecA* (*pe_pgrs35*) was disrupted in the CDC1551 strain by homologous recombination (25). We first examined the effect of this mutation on PE_PGRS proteins. We observed an increased molecular weight for some PE_PGRS proteins in the Δ*pecA* mutant as compared to the WT strain using immunoblot analysis (Fig. 5, lanes 1 and 2), indicating that PecA is active and processes PE_PGRS proteins in *M. tuberculosis*. Next, active PecA and catalytic inactive PecA^mut^ with a C-terminal HA-tag were expressed in *M. tuberculosis* WT and Δ*pecA* mutant strains, regulated by a theophylline-inducible riboswitch. The majority of PecA (expected weight ∼54 kDa) was detected around 55 kDa in both the WT and the Δ*pecA* mutant strain, while a minor fraction was observed around 68 kDa (lanes 3 and 4). The vast majority of PecA^mut^ detected in the Δ*pecA* mutant strain was visible ∼68 kDa (lane 6). Although PecA was observed at higher molecular weight than predicted, this data shows that the proteolytic activity of PecA is responsible for the processing of PecA, by the removal of ∼14 kDa of the N-terminus. Moreover, since only a fraction of PecA^mut^ was processed in the WT strain (lane 5), we suspect that PecA substrates are bound to inactive PecA preventing processing by endogenous active PecA. Another explanation could be that endogenous expression of *M. tuberculosis* PecA is too low to cleave all overexpressed PecA^mut^.

Complementation of the Δ*pecA* mutant with an intact *M. tuberculosis pecA* gene showed a similar PE_PGRS pattern as observed in the WT strain, whereas PE_PGRS proteins remained unprocessed following introduction of a gene coding for the PecA^mut^ active site mutant. This also suggests that PecA cleaves PE_PGRS proteins in addition to PecA. Moreover, an intermediate pattern could be observed after expression of PecA^mut^ in the WT strain, further suggesting that endogenously expressed PecA levels are low under laboratory conditions and/or that inactive PecA remains bound to the substrate, hindering active PecA from cleaving its substrate.

Next, we investigated whether *M. tuberculosis* PecA is, like its *M. marinum* orthologue, also involved in PPE processing. Therefore, we investigated molecular weights of *M. tuberculosis* PPE proteins we could detect with available antibodies. PPE38 plays a key role in secretion of PE_PGRS and PPE_MPTR proteins (12) and was suggested to be cleaved in the *M. tuberculosis* CDC1551 strain (26). Moreover, PPE38 was predicted as an *M. marinum* PecA substrate as two semi-tryptic peptides were enriched in the WT compared to the *pecA* deletion mutant (Table S3). In *M. tuberculosis* CDC1551 WT and Δ*pecA* mutant strains, we detected PPE38 at the predicted molecular weight ∼37 kDa and a band was also apparent ∼28 kDa, which was also observed previously (26). These results seem to indicate that PecA is not involved in the processing of PPE38 in *M. tuberculosis.* However, we also included overexpression of active PecA in these experiments. Upon overproduction of active PecA in WT cells and in the Δ*pecA* mutant strains, almost no PPE38 was detected. In contrast, introduction of the active-site mutant in WT and Δ*pecA* mutant strains resulted in higher levels of PPE38 compared to WT and Δ*pecA* mutant strains. Therefore, increasing the amount of active PecA present results in lower amounts of PPE38. We can conclude that PecA of *M. tuberculosis* is an active protease but is probably produced in low levels under laboratory conditions.

## Discussion

**Figure 6:**
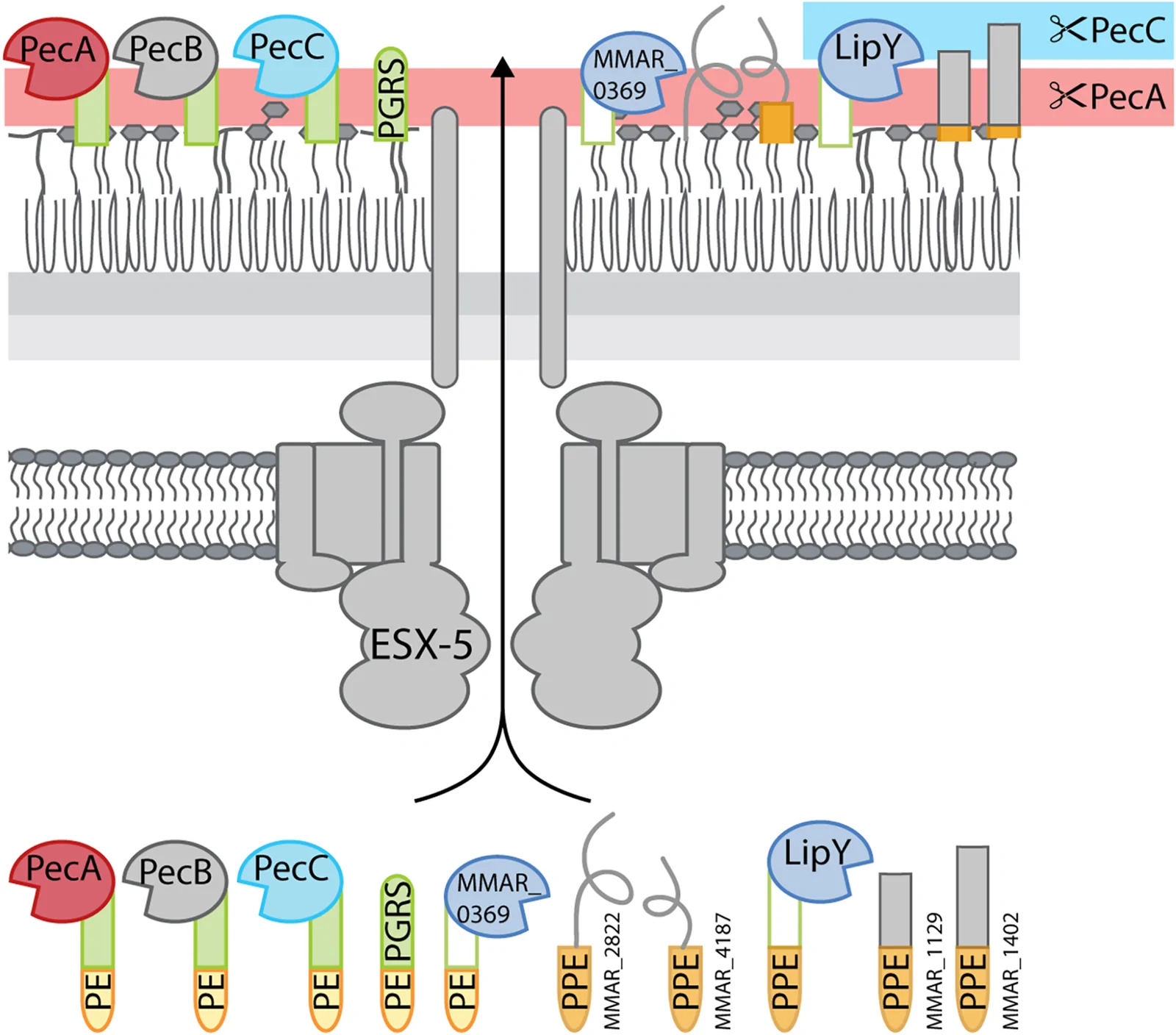
**Overview of PecA and PecC proteolytic activity in *M. marinum*.** After secretion through the ESX-5 system, PecA located on the mycobacterial surface cleaves a large number of PE_PGRS proteins including PecB, PecC and itself, by removing the PE domain. Of note, PecB was not detected by proteomics and was validated as a substrate only after overexpression. PecA was also demonstrated to process the PE protein MMAR_0369 and several members of the PPE protein family, including MMAR_2822, MMAR_4187, MMAR_1129 and MMAR_1402 and LipY. PecC was shown to process the PPE proteins MMAR_1129, MMAR_1402 and LipY, all of which are also validated PecA substrates in this study. Many other putative substrates were identified for PecA and PecC by proteomics, however these were not further validated in this study. Figure is adapted from (17).

Although PE/PPE proteins encode 10% of the *M. tuberculosis* genome, their characteristics such as high sequence homology and low number of trypsin cleavage sites (4, 27, 28) have complicated research to determine their functions. In recent years, significant effort has been devoted to studying their role. In this study, we investigated the (putative) aspartic proteases of *M. tuberculosis* and *M. marinum*, which are also PE_PGRS proteins. We confirmed the proteolytic activity of *M. tuberculosis* PecA and *M. marinum* PecC and discovered substrates for both in *M. marinum* (Fig. 6).

Previously, *M. marinum* PecA has been found to process various PE_PGRS proteins, including itself, as well as heterologously expressed *M. tuberculosis* PE protein and triacylglycerol lipase LipY (17). In this study, we identified additional PecA substrates, including aspartic proteases PecB and PecC, which are also PE_PGRS proteins. Using a label-free proteomics approach, we identified other surface substrates of PecA and PecB/PecC based on the analysis of semi-tryptic peptides of the mycobacterial surface fraction. Following validation experiments by determination of molecular weights of endogenous or overexpressed putative substrates in *M. marinum* WT and Δ*pecA* mutant strains, we found that MMAR_0369, a PE protein with cutinase function, was cleaved by *M. marinum* PecA at the C-terminal part of the PE domain. In addition to PE and PE_PGRS proteins, PecA was also shown to cleave the in *M. marinum* PPE protein LipY (MMAR_1547), the PPE protein MMAR_4187 in addition to PPE proteins that contain a major polymorphic tandem repeats (MPTR) domain of GxGNxG (2, 29), including MMAR_2822, MMAR_1129 and MMAR_1402. Semi-tryptic peptides analysis also predicted proteolytic activity by PecB and/or PecC on the mycobacterial surface, although substantially less than PecA. Following validation, 3 *M. marinum* PPE proteins (LipY, MMAR_1129 and MMAR_1402), which are also PecA substrates, were found to be cleaved by PecC.

Proteomics analysis of the potential PecA-generated peptides revealed a consensus cleavage site for PecA predominantly after an alanine residue. Interestingly, the PecA cleavage site was often within the YxxxD/E secretion motif at the end of the PE domain, where cleavage was predominantly observed in the YxxAE submotif. Previously, we reported that *M. tuberculosis* LipY can be cleaved at least 3 times in the presence of *M. marinum* PecA, where two cleavage sites were identified after the PE domain, containing 3 consecutive small amino acids like glycine and alanine, and one cleavage was predicted in the PE domain (17). One of these identified *M. tuberculosis* LipY cleavage sites matched with an enriched semi-tryptic peptide of *M. marinum* LipY in the presence of PecA in *M. marinum* containing a similar cleavage window. PecC was also observed to cleave *M. marinum* LipY, however this cleavage was not detected with proteomics. In a previous study, we suggested that PecA might be processed in or around the Type VII secretion motif (17). While a subset of the Type VII secretion motif in PE proteins was identified as a potential cleavage site, proteomic analysis failed to detect the semi-tryptic peptide of PecA that would confirm cleavage within this motif. On the other hand, semi-tryptic analysis did predict that PecC is processed in the Type VII secretion motif. Analysis of the semi-tryptic peptides could only confidently predict a cleavage site for PecA, possibly because of the larger amount of semi-tryptic peptides generated by PecA. Alternatively, since we analyzed all enriched semi-tryptic peptides, comparing the Δ*pecA* and *pecA*/*B*/C triple mutants could be obfuscating the results, as PecB and PecC may have different cleavage motifs.

Similar to the missing semi-tryptic peptide that belongs to processing of PecA itself, we hypothesize that more cleavages may be missed using our label-free proteomics approach. For example by low protein abundance or inability of detection by mass spectrometry for too small or large peptides caused by too many or few trypsin and/or protease cleavage sites. For instance, many PE and PPE proteins have very low numbers of trypsin cleavage sites. Moreover, processing of N-terminal domains might result in relocalization and release from the surface and therefore prevent peptide detection in the mycobacterial surface fraction. Another limitation of this approach is that selecting only leading razor peptides may restrict the ability to comprehensively identify all potential substrates. Finally, identification of substrates by comparing WT, Δ*pecA* and *pecA/B/C* triple mutant could be a limitation of this study. Possibly, substrates could be missed when sequential processing events occur, for example if substrates require first processing by PecA prior to further PecB/C processing events. Nevertheless, using our label-free proteomics approach, many putative substrates could be identified, for which a subset has been validated. Validation of putative substrates remains necessary as we observed that for the putative PecA substrate MMAR_2586 no products predicted to be generated by PecA (and PecC) were visible after overexpression of MMAR_2586 (Fig. S1*I*). Possibly, MMAR_2586 is fully degraded on the surface, which could be explained by the detection of 4 significant and 5 non-significant semi-tryptic peptides by proteomics after comparison of WT and *pecA/B/C* triple mutant strains.

Comparison of WT with the *ΔpecA* mutant revealed a notable set of semi-tryptic peptides that were absent in the WT but detected in the mutant. This pattern indicates that the absence of PecA alters proteolytic processing, for example by substrates that become available to alternative proteases, (for instance PecC) resulting in cleavage events that do not occur, or occur less efficiently, in WT cells.

In this study we also identified proteolytic activity of *M. tuberculosis* PecA, processing various PE_PGRS proteins including PecA itself, suggesting that PecA has a similar role in *M. tuberculosis.* Processing of PE_PGRS proteins was also observed previously after expression of *M. tuberculosis* PecA in *M. marinum* strains (17). However, our data suggest that inactive PecA^mut^ proteins remain bound to the substrate, hindering active PecA proteins from cleaving its unprocessed substrates. In *M. marinum ΔpecA* mutant expressing PecA^mut^, a high molecular weight protein is detected, which we hypothesize could also be a strong binding to a specific substrate (such as HA-tagged PecA). *M. tuberculosis* PPE38, which is involved in secretion of PE_PGRS and PPE_MPTR proteins (12), was found to be processed, and PecA was hypothesized to be responsible (26). Our data showed that cleaved PPE38 is also visible in *M. tuberculosis* CDC1551 *pecA* mutant strain, which shows that PecA is not involved in this processing event. Because PPE38 is nearly undetectable when active PecA is overexpressed, our data indicates that PecA promotes PPE38 degradation. The exact nature of this interaction, whether direct or indirect, is currently unknown. It is possible that the alanine-rich C-terminus of PPE38 serves as a target for cleavage, though this proposed mechanism warrants further study.

PecC may perform a similar biological role in *M. tuberculosis* as it does in *M. marinum*, however this is currently not investigated and remains to be explored, including the roles of the additional proteases present in *M. marinum* and *M. tuberculosis.* In our proteomics dataset, no tryptic or semi-tryptic peptides of *M. marinum* PecB were detected. Moreover, we did not observe proteolytic activity for PecB after validation of possible PecB or PecC substrates. Possibly, PecB is not present on the mycobacterial surface or expressed under the laboratory circumstances used for our experiments. In *M. tuberculosis* the gene encoding a third putative Pec aspartic surface protease, *pe_pgrs16*, was found expressed during all growth stages tested in *M. tuberculosis* Erdman strain. Moreover, mRNA expression was upregulated during nutrient depletion in culture, intracellularly in primary bone marrow macrophages and in mouse spleens following *in vivo* infection (30). In *M. tuberculosis* H37Rv, *pe_pgrs16* was upregulated during infection of human brain microvascular endothelial cells (31). This suggests that PE_PGRS16 plays a role during infection. On the other hand, this gene has a high number of sequence variations (102 of 200 *M. tuberculosis* clinical isolates tested) and 71 of these result in the loss of the C-terminal domain, including in *M. tuberculosis* CDC1551 strain (32). Previously, the crystal structure of the protease domain of PE_PGRS16 was solved, but no processing was observed (16). In conclusion, the function and activity of additional aspartic proteases PecB (*M*. *marinum*) and PE_PGRS16 (*M*. *tuberculosis*) remain unresolved and should be assessed in a native environment and in a late stage of infection.

In order to identify the function of PecA and PecC, it is necessary to understand the consequence of substrate processing, which may influence substrate function, activity or localization. The currently validated proteins belong to the PE/PPE protein families and are secreted by Type VII secretion system ESX-5 (5, 11). The functions of lipases MMAR_0369 and LipY are described, which are involved in cleavage of the non-natural substrates Tween-80 and triacylglycerol, respectively (23, 33). It was shown previously that processing of *M. tuberculosis* LipY in *M. marinum* did not affect lipase activity or differences in localization on the surface (17). However, the functions of the other validated substrates are currently unclear, and therefore, it is not known whether processing affects the protein function. Further research may validate more substrates and additionally can provide more evidence on the functions of substrates and fate of processing.

So far, *M. marinum* PecA and PecC cleavages identified appear predominantly in the N-terminal side of the validated PE/PPE substrates, which suggests that these PE and PPE domains function as transport peptides that are proteolytically removed following secretion. Although the roles of PE/PPE proteins in virulence remain largely unclear, since specifically PE domains and PPE proteins are found to be important immune epitopes (34, 35) and several PPE proteins have been used as antigens in promising vaccine developments (as reviewed in (36)) including the putative PecB or PecC substrate PPE18 (37), it could be speculated that the N-terminal domain of PE and PPE proteins are released to elicit a certain immune response or processed to evade immune recognition. On the other hand, current knowledge of PE_PGRS proteins and its role in virulence are conflicting, as several PE_PGRS proteins have been reported to contribute to virulence (15) but they are also found to be involved in immune response modulation and lowering inflammatory responses (12, 38, 39). Therefore, processing of PE_PGRS proteins and PPE_MPTR proteins might affect mycobacterial virulence and immune responses, which is in line with earlier results where *M. marinum* lacking PecA exhibited growth attenuation in zebrafish larvae (17). Finally, PecA may cleave proteins in the host, which is subject of further research. Our data suggest that release of the *M. marinum* PecA protease from the bacterial surface is limited, and that PecA activity is restricted on the surface of its own bacterial cell (Fig. S1*H*). Therefore, we hypothesize that possible host-protein cleavage by released PecA is also limited and relies on direct bacterial contact.

In conclusion, we identified substrates for *M. marinum* aspartic proteases using mass spectrometry and validated PecA and PecC proteolytic activity, which results in processing of several PE and PPE proteins. In addition, *M. tuberculosis* PecA cleaves PE_PGRS proteins including itself, suggesting a similar role for PecA in *M. tuberculosis.* However, the specific functions and significance of PecA and PecC-mediated processing of PE and PPE proteins during *Mycobacterium* infections remain to be elucidated.

## Experimental procedures

### Bacterial strains

All strains used in this study are listed in Table S2. *M. marinum* M (40) and *M. tuberculosis* CDC1551 (41) strains were cultured on plates containing Middlebrook 7H10 medium, OADC (Difco) and DelvoCid (200 μg/ml, DSM) or shaking at 90 rpm in Middlebrook 7H9 medium supplemented with ADC (Difco) and 0.05% Tween-80 (Sigma) at 30 °C. Cycloheximide (10 μg/ml, Sigma) was added to cultures of *M. tuberculosis* strains. *E. coli* NEB5α was grown on plates containing LB agar or in LB medium at 37 °C. When required, theophylline (0.5 mM, Sigma) was used for induction and appropriate antibiotics were added to culture plates or liquid cultures (50 μg/ml kanamycin (Sigma), 50 μg/ml hygromycin (Roche) or 30 μg/ml streptomycin (Sigma).

### Plasmid construction

To create pSMT3-PecA-HA, pSMT3-PecB-HA and pSMT3-PecC-HA, *MMAR_2933* (*pecA*), *MMAR_2272* (*pecB*) and *MMAR_1538* (*pecC*) were amplified by Phusion High Fidelity DNA polymerase (ThermoFisher) from *M. marinum M* genomic DNA using primers listed in Table S4. *PecA* and *pecC* PCR products were digested with restriction enzymes NheI and BglII and *pecB* PCR product was restricted with NheI and BamHI. All genes were ligated into the NheI- and BamHI-digested backbone pSMT3-LipYss-HA, replacing LipYss (42).

To create catalytic dead mutants of the aspartic proteases, PecA_D293G_, PecB_D241G_ and PecC_D234G_ mutations were made by site directed mutagenesis using overlap extension PCR with primers listed in Table S4. This generated the catalytic inactive constructs encoding for proteins called PecA^mut^, PecB^mut^, and PecC^mut^, respectively. Genes harboring active site mutations were ligated into the pSMT3 backbone as described above to create pSMT3-PecA-D293G-HA, pSMT3-PecB-D241G-HA and pSMT3-PecC-D234G-HA.

To construct plasmids for expression of predicted substrates, *M. marinum* genes *MMAR_1129*, *MMAR_2822*, *MMAR_4187* and *MMAR_2586* were amplified from *M. marinum* M genome using primers listed in Table S4 and cloned as described for construction of plasmid pSMT3-PecB-HA.

The CRISPRx plasmid containing hygromycin resistance gene (pCRISPRx-Sth1Cas9-L5-HygR) was constructed from pCRISPRx-Sth1Cas9-L5 (21). The hygromycin resistance gene was amplified by Phusion High Fidelity DNA polymerase (ThermoFisher) from plasmid pSMT3-PecA-D293G-HA using primers Hyg_Xho_FW and Hyg_RV. The purified PCR product was digested with XhoI and ligated into the XhoI- and EcoRV-digested pCRISPRx-Sth1Cas9-L5.

*M. tuberculosis* PecA (*pe_pgrs35*) was amplified from *M. tuberculosis* H37Rv genomic DNA using primers PE_PGRS35_EcoRI FW and PE_PGRS35-HA RV. A C-terminal HA-tag was introduced by a second PCR using HA-HindIII RV2. PST5552-PecAtb-HA was generated following restriction and insertion cloning *via* ligation of the EcoRI and HindIII PCR product into the HindIII- and EcoRI-digested pST5552-eGFP plasmid (Addgene 36255) containing a theophylline-inducible riboswitch. In order to express the active site mutant of *M. tuberculosis* PecA^mut^, D297G mutation was generated by site directed mutagenesis with overlap extension PCR and inserted into the pST5552 backbone as described above. Correct insertion of genes was confirmed by sequencing for all plasmids (Macrogen Europe).

For disruption of *M*. *tuberculosis pecA* (*pe_pgrs35*) by homologous recombination, pML2424-PecA was constructed as described previously (25). In short, upstream and downstream regions (Fig. S2) of *M. tuberculosis* CDC1551 *pecA* were amplified by PCR using iProof DNA polymerase (BioRad) and primers listed in Table S4. To prevent disruption of neighboring genes, 365 bp (5′) and 15 bp (3′) of the *pecA* gene remains present in the genome. PCR products were sequentially inserted into the pML2424 parental plasmid restricted with NsiI and PacI (upstream) and SwaI and SpeI (downstream) by In-Fusion cloning (Takara Bio). Correct insertions were confirmed by restriction digest. All plasmids used in this study are listed in Table S5 and were transformed into appropriate *M. marinum* and *M. tuberculosis* strains as described previously (21).

### Generation and identification of double and triple mutants by CRISPR-Cas9

All sgRNAs, except for the PecA sgRNA, were designed using CHOPCHOP (22). Single frameshift mutants were identified as described previously (21). Double and triple *M. marinum* mutants were generated by sequential transformation of CRISPR-Cas9 plasmids and identification of frameshift mutants. Briefly, the pCRISPRx-Sth1Cas9-L5-KanR plasmid (Addgene 140993) in the identified single mutant strain was replaced with pCRISPRx-Sth1Cas9-L5-HygR containing the sgRNA targeting the appropriate second target gene (Table S2). In all final mutants, CRISPR-Cas9 plasmid was replaced by the integrative plasmid pTdTomato-L5 ((21), Addgene 140994).

### Generation of *ΔpecA* deletion mutant in *M. tuberculosis*

Genomic *M. tuberculosis* CDC1551 *pecA* (*pe_pgrs35*) was disrupted as described previously (25). In short, *M. tuberculosis* CDC1551 WT strain was transformed with the temperature-sensitive plasmid pML2424-PecA and transformants were selected on 7H10 plates containing hygromycin. After incubation at 37 °C, separate GFP & RFP positive colonies were grown until OD_600_ ∼1 in 7H9 medium prior to plating on 7H10 plates containing 2% sucrose and incubation at 40 °C for 3 to 4 weeks. GFP-positive RFP-negative colonies *pecA::hygR*, *gfp* mutants were confirmed as double cross over cells by PCR and restriction digest with PacI (Fig. S2).

### Surface protein extraction and sample preparation for proteomics

*M. marinum* wildtype, Δ*pecA*, and *pecA/B/C* triple frameshift mutant containing pTdTomato-L5 were cultivated in 7H9 medium containing 0.2% glycerol, ADC and 0.05% Tween-80 at 30 °C. One day prior to extraction of surface proteins, bacteria were washed two times in 7H9 containing 0.2% glycerol and 0.2% dextrose and grown in 100 ml starting from OD_600_ of 0.35. After growth overnight, the culture was washed with PBS and incubated with 0.5% Genapol X-080 supplemented with complete protease inhibitor cocktail (Roche) head-over-head for 30 min at room temperature. Extracted surface proteins were separated from cells by centrifugation and supernatants were filtered through 0.2 μm filters. Surface proteins were precipitated overnight on ice with tRNA and 100% TCA in acetone, washed two times in acetone and air-dried. Next, protein was solubilized in urea buffer (8M urea in 100 mM ammonium bicarbonate (ABC)). Samples were centrifuged and protein concentration of soluble protein (supernatant) was measured using Detergent Compatible Protein Assay (BioRad). Per sample, 50 μg of protein was reduced for 1h at room temperature by addition of TCEP (Tris(2-carboxyethyl) phosphine) in a final concentration of 5 mM and alkylated with iodoacetamide in the dark at a final concentration of 5.5 mM. Subsequently, samples were cleared of Genapol X-080 in samples using Filter Aided Sample Preparation (FASP). Briefly, samples were added Microfuge Amicon 10 kDa cut-off spin filters (Milipore) and centrifuged at 14,000*g* for 15 min (15 °C). Next, 6 washing steps were performed by adding 400 μl urea buffer to the filter and centrifuging at 14000*g* for 15 min, prior to 3 washes with 50 mM ABC. Finally, 400 μl of 50 mM ABC was added to the filter in addition to 1 μg of modified sequencing-grade trypsin (Promega, V5111, 1:50 trypsin to protein ratio) and incubated for 18h in a humidified chamber at 37 °C. Peptides were captured following centrifugation at 14000*g* for 15 min, including the flowthrough after addition of 250 μl of 100 mM NaCl to the filter and centrifugation at 14000*g* for 15 min. Peptides were concentrated in a vacuum-concentrator and desalted with OASIS HLB Cartridges. Growing cultures and extracting protein was carried out in three biological replicates.

### LC-MS/MS

Peptides were separated by an Ultimate 3000 nanoLC-MS/MS system (Dionex LC-Packings) equipped with a 45 cm × 75 μm ID fused silica column custom packed with 1.9 μm 120 Å ReproSil Pur C18 aqua (Dr Maisch GMBH). After injection, peptides were trapped at 6 μl/min on a 10 mm × 100 μm ID trap column packed with 5 μm 120 Å ReproSil Pur C18 aqua in 0.05% formic acid. Peptides were separated at 300 nl/min in a 10 to 40% gradient (buffer A: 0.5% acetic acid (Fisher Scientific), buffer B: 80% ACN, 0.5% acetic acid) in 60 min (100-min inject-to-inject). Eluting peptides were ionized at a potential of +2 kVa into a Q Exactive mass spectrometer (Thermo Fisher). Intact masses were measured at resolution 70,000 (at m/z 200) in the orbitrap using an AGC target value of 3E6 charges. The top 10 peptide signals (charge-states 2+ and higher) were submitted to MS/MS in the HCD (higher-energy collision) cell (1.6 amu isolation width, 25% normalized collision energy). MS/MS spectra were acquired at resolution 17,500 (at m/z 200) in the orbitrap using an AGC target value of 1E6 charges, a maxIT of 60 ms, and an underfill ratio of 0.1%. Dynamic exclusion was applied with a repeat count of 1 and an exclusion time of 30 s.

### Mass spectrometry data analysis

MS/MS spectra were searched against a *M. marinum* M reference proteome FASTA file (5418 entries) using MaxQuant 1.6.10.43 (Cox J & Mann M, 2008). Enzyme specificity was set to semi-specific, in order to capture peptides that were both cut by trypsin and another protease. Cysteine carboxyamidomethylation (Cys, +57.021464 Da) was treated as fixed modification and methionine oxidation (Met, +15.994915 Da) and N-terminal acetylation (N-terminal, +42.010565 Da) as variable modifications. Peptide precursor ions were searched with a maximum mass deviation of 4.5 ppm and fragment ions with a maximum mass deviation of 20 ppm. Peptide and protein identifications were filtered at an FDR of 1% using the decoy database strategy. Global normalization to the total count of each sample was performed. The minimal peptide length was seven amino acids. Differential abundance was assessed using the Limma package, employing empirical Bayes statistics for the analysis of intensity-based quantification data (43). To account for multiple hypothesis testing, *p*-values were adjusted using the Benjamini-Hochberg procedure. For the generation of the volcano plots Missing Not At Random (MNAR) data were imputed with the following values: down-shift (log_2_ units) was set to 1.8 and imputation width (sd) was set to 0.3. Valid-value filtering was applied by requiring a minimum of two valid (non-missing) LFQ intensity values in at least one of the comparison groups.

Peptides preceding or ending with arginine (R) or lysine (K) were classified as semi-tryptic peptides. Cleavage windows of semi-tryptic peptides were created using Excel and the amino acid probabilities at each position were analyzed using the probability logo generator (pLogo) (44), using all coding sequences (CDS), or only PE and PPE FASTA CDS of *M. marinum* M (NCBI) as background. Volcano plots were generated using R and R studio version 4.2.1 (Code repository (45)). The proteomics data are available *via* ProteomeXchange with identifier PXD067990.

### Peptide synthesis and purification

Peptide Synthesis: the FRET peptide FP53 [Dabcyl]-QVPPFAAAQ-[K(FITC)]-NH_2_ (Fig. S3) was synthesized *via* automated Fmoc-based solid-phase peptide synthesis (SPPS) using a Syro I synthesizer (MultiSynTech GmbH). Synthesis was performed on H-Rink amide ChemMatrix resin (10–100 μmol scale). Amino acid couplings were executed using a double-coupling strategy: 1) 4 eq. PyBOP/Oxyma/DIPEA (40 min) and 2) 4 eq. HATU/Oxyma/DIPEA (40 min) in DMF. Fmoc deprotection was conducted using 25% piperidine in DMF (v/v), followed by a capping step with Ac_2_O/DIPEA/DMF (1:1:8) for 10 min. Fluorophore Incorporation: following N-terminal deprotection, the Dabcyl moiety was incorporated using 4 eq. each of Dabcyl acid, COMU, and Oxyma with 8 eq. DIPEA in DMF (2 × 30 min). To facilitate C-terminal labeling, an N-α-Fmoc-N-ε-MMT-L-lysine was integrated as the initial residue. Post N-terminal modification, the MMT group was selectively removed using 1% TFA and 2% TIPS in DCM. FITC (4 eq.) was then conjugated to the lysine sidechain in the presence of DIPEA (6 eq.) in DMF (2 × 1.5 h). Purification and Characterization: peptides were cleaved from the resin and globally deprotected using a cocktail of TFA/H_2_O/TIPS (94:2.5:1, v/v/v) for 3 h. Crude peptides were precipitated in cold diethyl ether, lyophilized, and purified by semi-preparative RP-HPLC (Agilent 1100) on a C18 column (Macherey-Nagel Nucleodur, 10 × 125 mm, 5 μm) using an ACN/H_2_O gradient (+0.1% TFA). Final purity and identity were confirmed by analytical LC-MS (Agilent 1260 Infinity/6120 Quadrupole) with electrospray ionization (Fig. S3). Concentration Determination: stock concentrations of the purified FRET peptide were determined by UV-Vis spectroscopy in 100 mM sodium phosphate buffer (pH 8.5). Subsequently, the absorption at 494 nm was measured. The extinction coefficients of both fluorophores were considered in the calculation of the concentration, following the Lambert-Beer law (ε(FITC)494 = 77,000 M^−1^ cm^−1^, ε(Dabcyl)494 = 14,000 M^−1^ cm^−1^) (46).

### Fluorescence resonance energy transfer (FRET)-based kinetic protease activity assay

*M. marinum* strains were grown in 7H9 medium supplemented with ADC and 0.05% Tween-80 at 30 °C. One day prior to the kinetic measurement, cells were inoculated at an OD600 of 0.4 and grown overnight in the dark at 30 °C. A bacterial suspension of 1∗10^8^ CFU/ml was prepared of each strain (1 OD unit is calculated as 2∗10^8^ CFU/ml). Bacterial suspensions (25 μl) were mixed with 10 μM FP53 (25 μl) peptide in μClear Chimney black clear-bottom sterile 96-well plates (Greiner Bio-One), to obtain final concentrations of 0,5∗10^8^ CFU/ml and 5 μM FP53. FP53 peptides were incubated with bacterial strains at 30 °C in the BioTek Synergy H1 plate reader with 3 mm continuous linear shaking. OD600 and fluorescence (excitation 485 nm, emission 528 nm) was measured every 15 min for 90 min followed by every 30 min for 4 h. Per experiment, every strain was tested in duplicates. The average of t = 0 was subtracted from the averages of all timepoints and further normalized by the average of FP53 at the corresponding timepoint. Relative fluorescence was determined by normalizing to the WT at the final time point. Medium and medium containing DMSO served as negative controls.

### Sample preparation for immunoblotting

For preparation of whole-cell lysates, *M. marinum* strains were either cultivated in 7H9 supplemented with ADC and Tween-80 and washed in PBS when they reached an OD600 ∼1, or cells were deposited directly in PBS from 7H10 culture plates. Afterwards, cells were subjected to bead beating for 2 min with 0.1 mm Zirconia silicon beads (Biospec Products), and boiled for 10 min at 96°C after addition of 5x SDS sample buffer (312.5 mM Tris-HCl pH 6.8, 10% SDS, 30% glycerol, 500 mM DTT and 0.02% bromophenol blue).

For secretion analysis, cells were cultured in 7H9 containing 0.2% glycerol, ADC and 0.05% Tween-80 at 30 °C. One day prior to separating bacterial secretion fractions, bacteria were washed two times in 7H9 containing 0.2% glycerol and 0.2% dextrose and cultured from starting OD600 0.4. The next day, culture supernatant was separated from bacteria by centrifugation, filtered through a 0.2 μm filter and precipitated with tRNA and TCA. Bacterial cells were washed with PBS, and a fraction of cells was used for preparation of whole-cell lysates, while another fraction was incubated head-over-head with Genapol X-080 for 30 min at room temperature. Genapol supernatants containing surface proteins were separated from Genapol pellets by centrifugation. After washing in PBS, Genapol pellet fraction as well as whole cell lysate fractions were subjected to beadbeating for 2 min. SDS sample buffer was added to all samples prior to boiling at 96 °C for 10 min.

### SDS PAGE and immunoblotting

Samples were separated using SDS PAGE gels and imaged with Coommassie G250 staining or transferred onto nitrocellulose membranes (Amersham) and stained with Ponceau S (0.1% in 5% acetic acid) to examine loading equality. Membranes were blocked with 5% milk in PBST, incubated with primary antibody (anti-HA (1:5000; Biolegend 901503), anti-GroEL2 (CS44; Colorado State University), anti-PGRS monoclonal 7C4.1F7 (5), anti-PPE38 (26) or polyclonal antibody against the C-terminal part of MMAR_1402 (Weerdenburg *et al*., unpublished results)) for 1h shaking at room temperature or overnight at 4 °C. Following washing with PBST and incubation with secondary goat anti-rabbit (Rockland 611-1302) or goat anti-mouse peroxidase-conjugated IgG (American Qualex Antibodies A109PS) antibodies for 1h at room temperature and subsequent washing steps, blots were imaged with ECL Prime Western Blotting Detection Reagents (Amersham) and the Amersham Imager 600.

## Data availability

Code repository (45). Also data set ProteomeXchange accession: PXD067990 is available via https://www.ebi.ac.uk/pride/archive/projects/PXD067990.

## Supporting information

This article contains supporting information (17, 20, 21, 23, 40, 41, 42, 45, 48).

## Conflict of interest

The authors declare that they have no conflicts of interest with the contents of this article.
