# Supplementary material for: Multiple aspartic proteases process PE and PPE proteins on the mycobacterial surface of pathogenic mycobacteria
Source: J Biol Chem. 2026 Aug 13;302(10):113450. doi: 10.1016/j.jbc.2026.113450 (PMC13578627; doi:10.1016/j.jbc.2026.113450)
Supplement: Supplementary file 1 [file mmc1.pdf]

Supporting Information

Title

Multiple aspartic proteases process PE and PPE proteins on the mycobacterial surface of pathogenic mycobacteria

Authors

Aniek S. Meijers[1], James L. Gallant[2], Alexander Speer[3], Janneke Maaskant[3], Saara Hietamäki[3], Roy Ummels[3], Felix M. Paulußen[2,4], Tom N. Grossmann[4], Thang V. Pham[5], Sander R. Piersma[5], Connie R. Jiménez[5], Sergey Nejentsev[6,7], Wilbert Bitter[2,3], Coen P. Kuijl[3]

Affiliations

[1]*Medical Microbiology and Infection Control, Amsterdam UMC, Vrije Universiteit Amsterdam, Cancer Center Amsterdam, Amsterdam, the Netherlands*

[2]*Molecular Microbiology, Vrije Universiteit Amsterdam, Amsterdam, the Netherlands*

[3]*Medical Microbiology and Infection Control, Amsterdam UMC, Vrije Universiteit Amsterdam, Amsterdam Institute of Infection & Immunity, Amsterdam, the Netherlands*

[4]*Department of Chemistry and Pharmaceutical Sciences, Vrije Universiteit Amsterdam, Amsterdam, the Netherlands*

[5]*Proteomics Core Resource, Laboratory Medical Oncology, Amsterdam UMC, Vrije Universiteit Amsterdam, Amsterdam, the Netherlands*

[6]*Department of Medicine, University of Cambridge, Cambridge, United Kingdom*

[7]*Department of Molecular Cell Biology and Immunology, Amsterdam University Medical Centers, Amsterdam, the Netherlands*

Supporting Figures

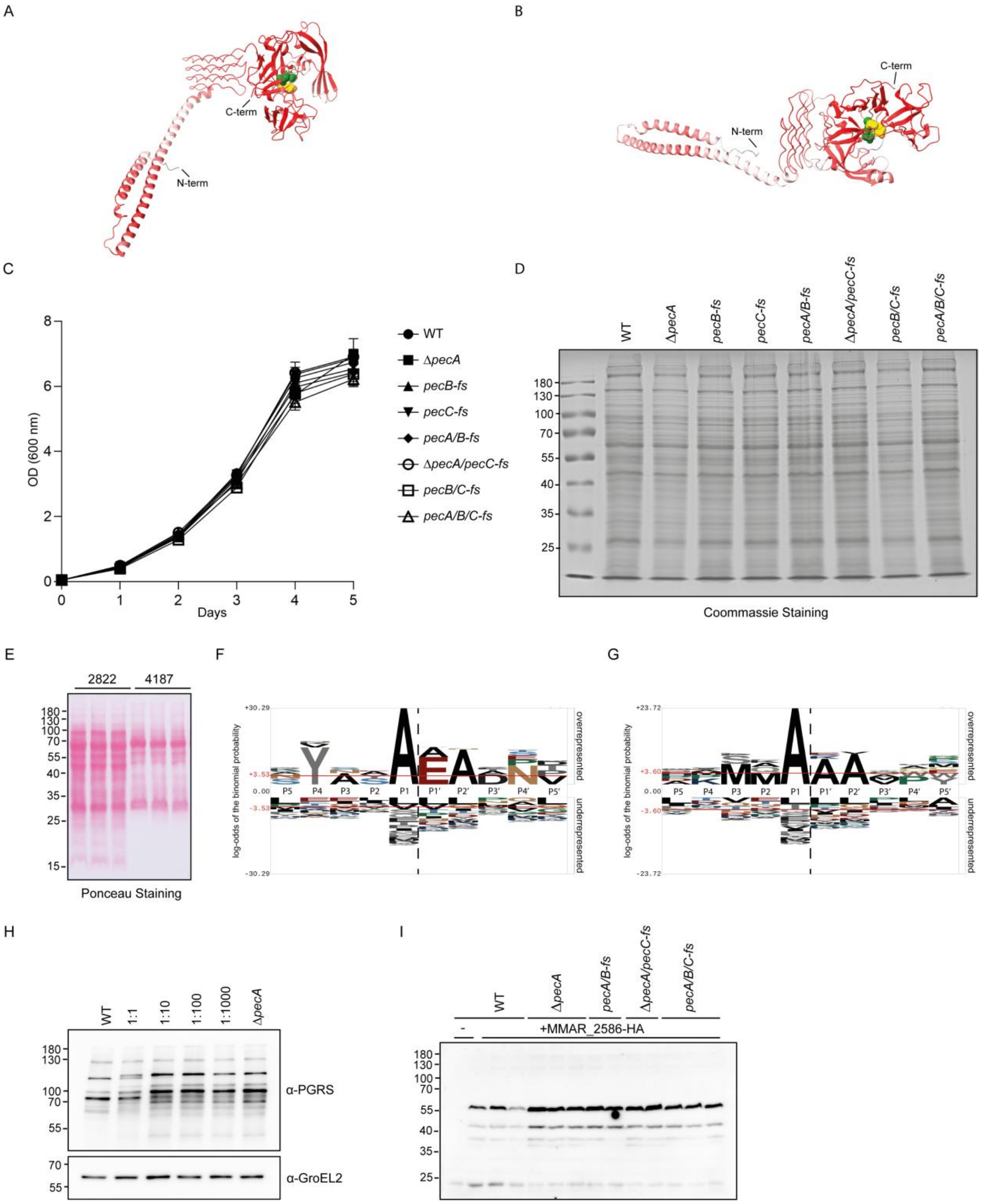

Figure S1. (A) Alphafold2 prediction of PecB (MMAR_2272; AF-B2HPB7-F1-v6) and (B) Alphafold2 prediction of PecC (MMAR_1538; AF-B2HGK3-F1-v6) [20]. B-factor was visualized using UCSF ChimeraX v1.11.1, which represents the predicted Local Distance Difference Test (pLDDT) scores, with high confidence scores in red and low confidence scores in blue. DTG and DSG motifs were colored green and yellow, respectively, and residues with side chains are shown as spheres. (C) Optical density at 600 nm of *M. marinum* WT and single, double and triple *pecA*, *pecB* and *pecC* mutant strains in 7H9 medium was measured for 5 days. Mean and SD larger than symbol sizes are shown. (D) Whole cell lysates of *M. marinum* WT, single, double and triple *pecA/B/C* mutant strains after culture in 7H9 medium, stained by Coommassie G250. Data is representative of 2 biological replicates. (E) Ponceau staining of the immunoblots shown in Figure 3B-C to confirm equal loading of cell lysates of *M. marinum* WT, Δ*pecA* mutant and *pecA/B/C* triple mutant expressing MMAR_2822-HA (Figure 3B) and MMAR_4187-HA (Figure 3C). (F) PecA cleavage motif identified through analysis of 70 cleavage windows derived from enriched semi-tryptic peptides of PE proteins, predicted by pLogo using *M. marinum* PE and PPE FASTA CDS as background. (G) PecA cleavage motif within PPE proteins by analysis of 52 cleavage windows with *M. marinum* PE and PPE FASTA CDS as background. (H) WT and Δ*pecA* mutants strains were grown in 7H9 medium and co-cultured in 1:1, 1:10, 1:100 or 1:1000 ratios for 20-24 hours. Whole cell lysates were prepared and PE_PGRS proteins were detected by immunoblot (0.3 OD units were detected per lane). Data is representative of 2 biological replicates. (I) *M. marinum* WT, Δ*pecA*, *pecA/B, pecA/C* and *pecA/B/C* triple mutant expressing MMAR_2586-HA. Samples were taken from cells grown on 7H10 plates.

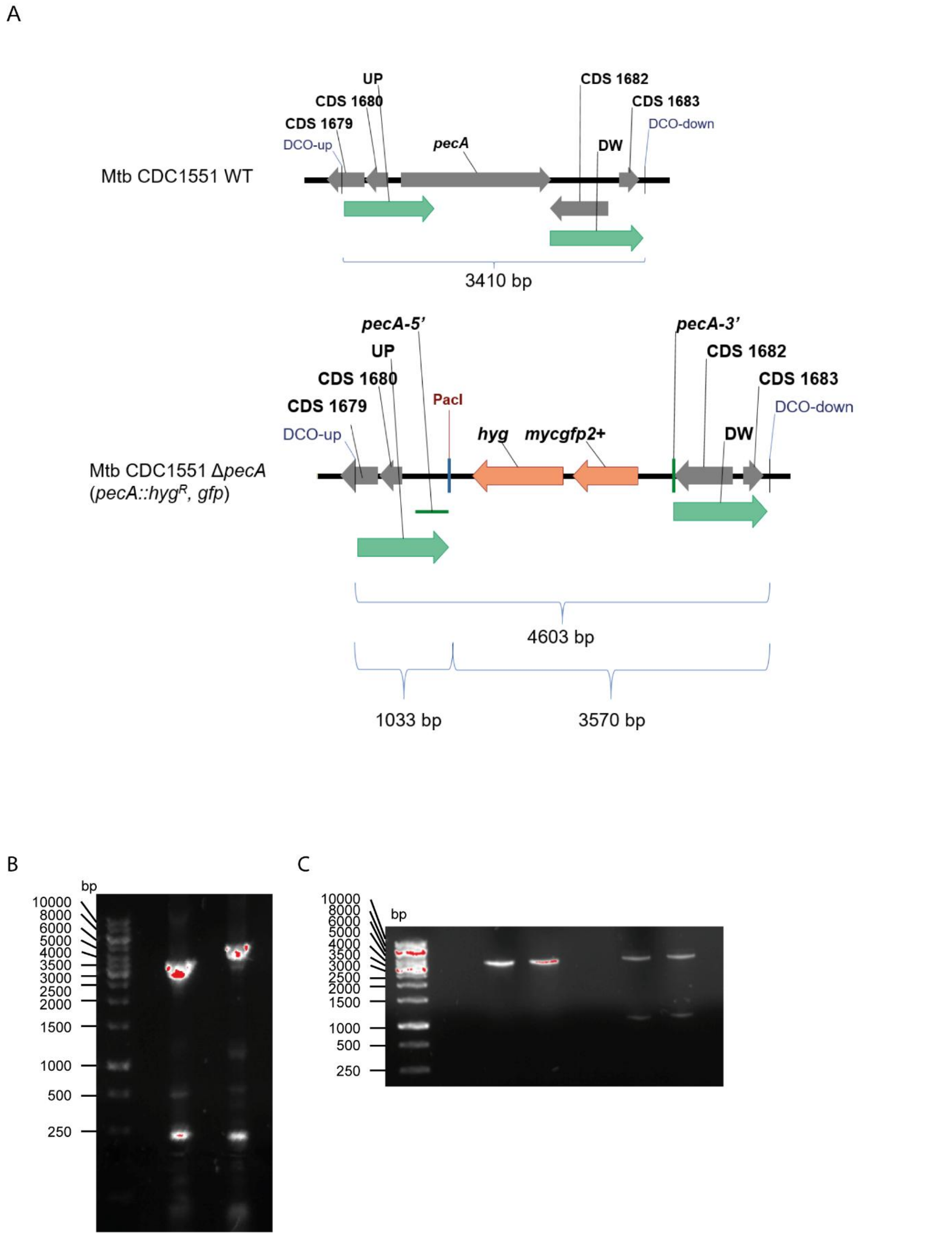

Figure S2. (A) Schematic overview of *M. tuberculosis* CDC1551 *pecA* locus found in WT and the Δ*pecA* mutant. Homologous regions used for recombination are indicated by green arrows, residual *pecA* sequence is indicated with green lines and primer binding sites are depicted in blue. PacI restriction site is shown in red. (B) Validation of homologous recombination by PCR using primer pair DCO up and DCO down (Table S4). (C) PacI digestion of 3410 bp and 4603 bp PCR products from (B) to validate replacement of *pecA.*

A

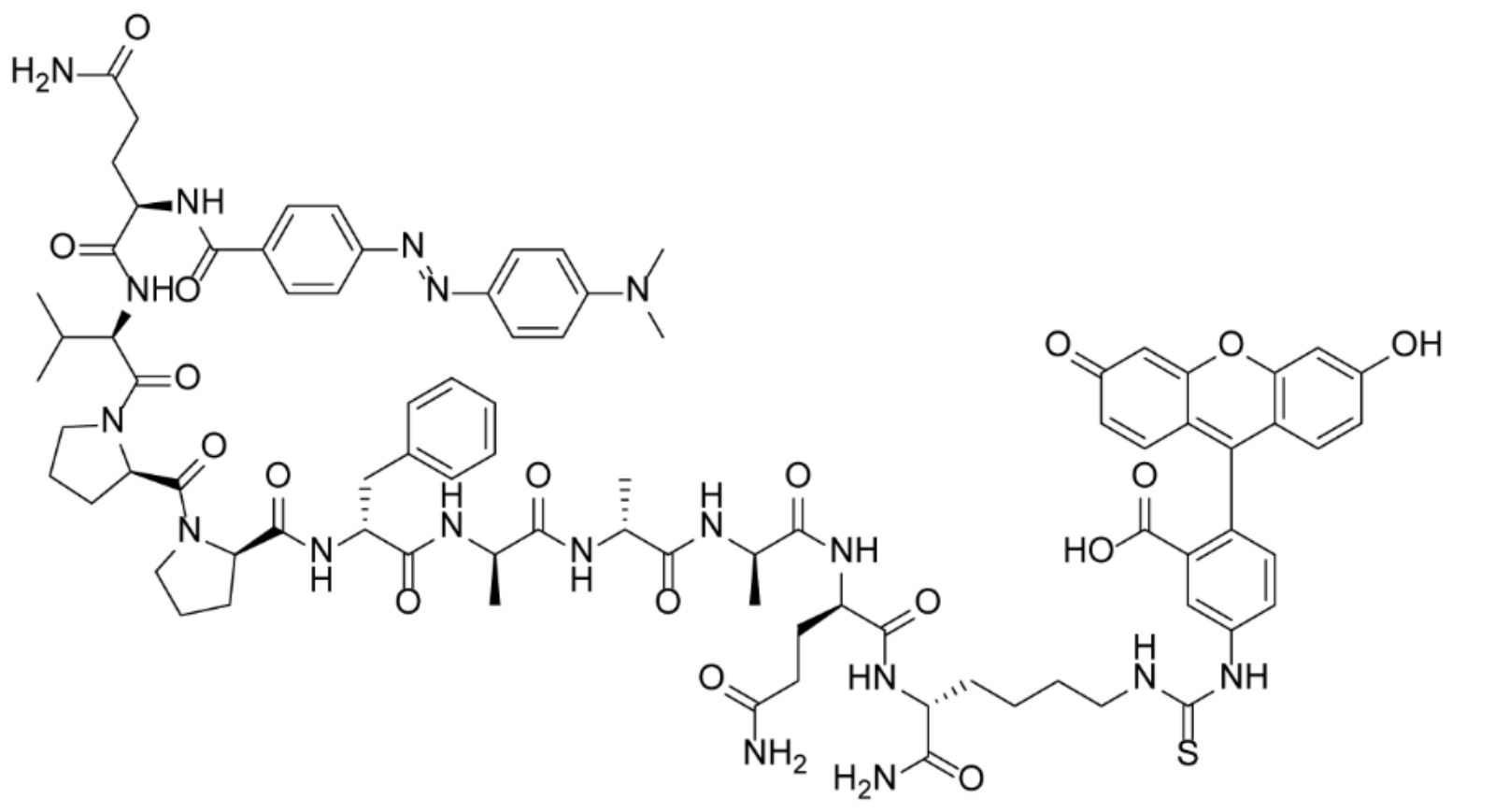

B

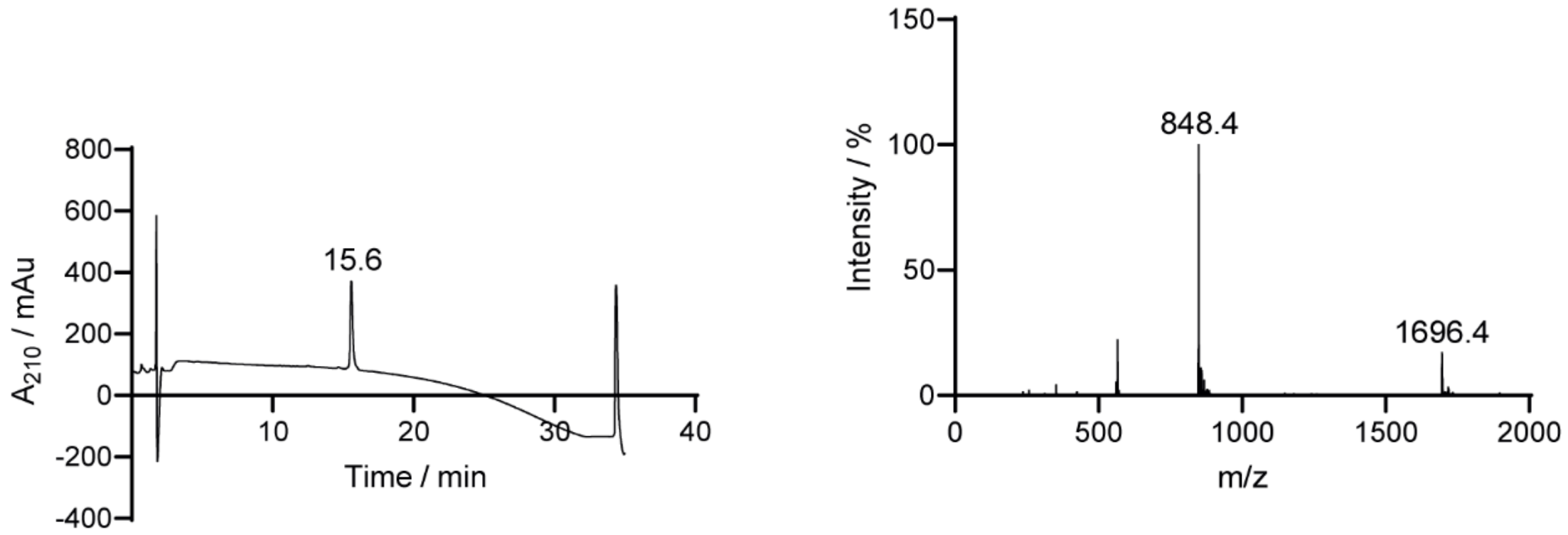

Figure S3. (A) Chemical structure of FRET peptide FP53 [DABCYL]QVPPFAAAQ[K-FITC]-NH2 containing a N-terminal Dabcyl and C-terminal FITC. (B) Analytical analysis of FP53: Left: HPLC chromatogram at λ = 210 nm, peak retention time: 15.6 min, gradient: 5-95 % ACN in 30 min; Right: MS spectrum, MW: 1695.92 g·mol^{-1}, calc. m/z: 1695.7 / 848.4.

Supporting Tables

	Domain	PecA		PE_PGRS16 & PecB		PecC	
	Similarities/ Identities	PE_PGRS35	MMAR_2933	PE_PGRS16	MMAR_2272	PE26	MMAR_1538
PecA	PE_PGRS35		82	58	52	53	57
	MMAR_2933	69		56	52	53	56
	PE_PGRS16	45	43		60	57	59
	MMAR_2272	40	38	45		55	56
PecC	PE26	39	38	43	38		82
	MMAR_1538	44	43	48	43	71	

Table S1. Amino acid sequence comparison of protease domains of *M. tuberculosis* and *M. marinum* aspartic proteases PecA (PE_PGRS35/MMAR_2933), PecB (MMAR_2272), PE_PGRS16 and PecC (PE26/MMAR_1538) using the Basic Local Alignment Search Tool (NCBI). Upper triangle: Values represent the percentage of amino acid similarity. Lower triangle: Values represent the percentage of amino acid identity.

Table S2. Strains and mutants used in this study

**Strain**	**Description/Frameshift mutation**	**Parental strain**	**Reference**
E.coli NEB 5-alpha	For molecular cloning	-	New England Biolabs
M. marinum M wildtype	Wildtype strain	-	[40]
M. marinum ΔpecA mutant	Deletion mutant	*M. marinum* M	[17]
M. marinum pecB frameshift mutant	c.75_90del	*M. marinum* M	[21]
M. marinum pecC frameshift mutant	c.52_53insT	*M. marinum* M	[21]
M. marinum pecA/B double frameshift mutant	*pecA* c.17_18del<br>*pecB* c.17_18del	*M. marinum* M	[21]
M. marinum pecA/C double (frameshift) mutant	*ΔpecA* deletion mutant<br>*pecC* c.52_53insG	*M. marinum ΔpecA*	This study
M. marinum pecB/C double frameshift mutant	*pecB* c.75_90del<br>*pecC* c.44_51del	*M. marinum pecB* frameshift mutant	This study

M. marinum *pecA/B/C* triple frameshift mutant	*pecA* c.17_18del *pecB* c.17_18del *pecC* c.51_52insC	*M. marinum pecA/B* double frameshift mutant	This study
M. tuberculosis CDC1551 wildtype	Wildtype strain	-	[41]
M. tuberculosis CDC1551 *ΔpecA* mutant	(*pecA::hyg^R*, *gfp*) (See Figure S2)	*M. tuberculosis* CDC1551	This study

Table S3. Mass spectrometry dataset. The script including a preformatted data table used to analyse the data can be found here: https://doi.org/10.5281/zenodo.20209202 [45].

Table S4. Primers used in this study.

**Primer name**	**Primer sequence (5' --> 3')**
MMAR_2933_NheI FW	AAAGCTAGCGTGTCGCTTCTGGTGGTGGC
MMAR_2933_BglII_RV	TTTAGATCTGGGCATATCAAAGACCGTCG
MMAR_2272_NheI_FW	AAAGCTAGCGTGTCTCTTCTGGTGGTGGC
MMAR_2272_BamHI_RV	AAAGGATCCGAAGTCAAAAATTGTCTGCCCA
MMAR_1538_NheI_FW	AAAGCTAGCGTGCGCGCCGAGCAAACCGT
MMAR_1538_BglII_RV	TTTAGATCTCGGCGGCGGGTAGTTGAATA
MMAR_2933_D293G_Fw	CGGTTCTGATTGGCACCGGATCCGC
MMAR_2933_D293G_Rv	GCGGATCCGGTGCCAATCAGAACCG
MMAR_2272_D241G_Fw	CCGTGACGGTCGGTACCGGAGCCAG
MMAR_2272_D241G_Rv	CTGGCTCCGGTACCGACCGTCACGG
MMAR_1538_D234G_Fw	CTGTGCTGCTTGGCACCGGATCTGC
MMAR_1538_D234G_Rv	GCAGATCCGGTGCCAAGCAGCACAG
MMAR_4187 FW NheI	AAAGCTAGCATGACCTATCCCATGTTTCC
MMAR_4187 Rv BamHI	TTTGGATCCCATCGTGGGTAGCACCGTCA
MMAR_2822 FW NheI	AAAGCTAGCATGAACTTCTCCGTGCTTCC
MMAR_2822 RV BamHI	TTTGGATCCCGGGGTGACGCTCTGGAAGA
MMAR_1129 FW NheI	AAAGCTAGCATGACGAACTTTGCAATGTT
MMAR_1129 RV BamHI	TTTGGATCCGGACTTGATGGCCAGCTTGC
MMAR_2586 Fw NheI	AAAGCTAGCATGTCGACGATTCATCGACT
MMAR_2586 RV BamHI	TTTGGATCCTGCCAGTTCCGTGTGCATCA
Hyg_XhoI_FW	AAACTCGAGCTACGGGGTCTGACGCTCAG
Hyg_RV	TTTCGACTGTCCTCGTTGATCCTT
PE_PGRS35_EcoRI FW	AAAGAATTCGTGTCATTTCTGGTCGTGGT
PE_PGRS35-HA RV	GGCACGTCGTACGGGTAAGATCCCGCCGGATGATCAAAGA

HA-HindIII RV2	CTAAAGCTTACGCGTAGTCCGGCACGTCGTACGGGTA
PE_PGRS35-D297G-Fw	GTCAACCATTCTGGTCGGAACCGGATCCGCTGGTC
PE_PGRS35-D297G-Rv	GACCAGCGGATCCGGTTCCGACCAGAATGGTTGAC
pML-PecA Up FW	AGACAATAACCATGCATATGCTCGGCGGTGAAAGATG
pML-PecA Up RV	ACGAAGTTATTAATTAAATTAGCGGACGCCCCAACAA
pML-PecA Down FW	TATACGAAGTTATTTAAATGATCATCCGGCGTGATCGAGC
pML-PecA Down RV	AATATTGATCCACTAGTTGCTGGACCCGCTTCCTCAA
DCO up	AAAGGCTCGTTGCGTGGCAG
DCO down	CCGACGCCAGCATGTTGTTGTG

Table S5. Plasmids used in this study.

**Plasmid**	**Description**	**Reference**
pSMT3-PecA-HA	pSMT3 backbone, *hsp60* promotor, *MMAR_2933* insert, C-terminal HA tag, HygR	This study
pSMT3-PecB-HA	pSMT3 backbone, *hsp60* promotor, *MMAR_2272* insert, C-terminal HA tag, HygR	This study
pSMT3-PecC-HA	pSMT3 backbone, *hsp60* promotor, *MMAR_1538* insert, C-terminal HA tag, HygR	This study
pSMT3-PecA-D293G-HA	pSMT3 backbone, *hsp60* promotor, *MMAR_2933* insert with D293G mutation, C-terminal HA tag, HygR	This study
pSMT3-PecB-D241G-HA	pSMT3 backbone, *hsp60* promotor, *MMAR_2272* insert with D241G mutation, C-terminal HA tag, HygR	This study
pSMT3-PecC-D234G-HA	pSMT3 backbone, *hsp60* promotor, *MMAR_1538* insert with D234G mutation, C-terminal HA tag, HygR	This study
pSMT3-MMAR_1129-HA	pSMT3 backbone, *hsp60* promotor, *MMAR_1129* insert, C-terminal HA tag, HygR	This study
pSMT3-MMAR_2822-HA	pSMT3 backbone, *hsp60* promotor, *MMAR_2822* insert, C-terminal HA tag, HygR	This study
pSMT3-MMAR_4187-HA	pSMT3 backbone, *hsp60* promotor, *MMAR_4187* insert, C-terminal HA tag, HygR	This study
pSMT3-mmLipY-HA	pSMT3 backbone, *hsp60* promotor, *MMAR_1547* insert, C-terminal HA tag, HygR	[48]
pSMT3-MMAR_0369-HA	pSMT3 backbone, *hsp60* promotor, *MMAR_0369* insert, C-terminal HA tag, HygR	[23]

pSMT3-MMAR_2586-HA	pSMT3 backbone, *hsp60* promotor, *MMAR_2586* insert, C-terminal HA tag, HygR	This study
pSMT3-hsp60	pSMT3 backbone, *hsp60* promotor, HygR	[42]
pCRISPRx-Sth1Cas9-L5-KanR	Sth1Cas9, sgRNA TetR, KanR, L5Int,	[21]
pCRISPRx-Sth1Cas9-L5-HygR	Sth1Cas9, sgRNA, TetR, HygR, L5Int,	This study
pTdTomato-L5	pMV361 backbone *hsp60* promoter, *tdTomato,* StrepR	[21]
pST5552-eGFP	pMV261 based vector, *KanaR*, theophylline-responsive riboswitch, *gfp*	Addgene 36255
pST5552-PecAtb-HA	pMV261 based vector, KanaR, theophylline-responsive riboswitch, *PE_PGRS35,* C-terminal HA tag	This study
pST5552-PecAtb-D297G-HA	pMV261 based vector, KanaR, theophylline-responsive riboswitch, *PE_PGRS35* with D297G mutation, C-terminal HA tag	This study
pML2424-PecA	Thermosensitive Pal5000, pUC-origin, *sacB, sacR, hygR, gfp, tdTomato*, homologous regions *pe_pgrs35* upstream and downstream	This study
